# Probing the Interface of Plasma, Metal, and Catalyst Support in Plasma-Assisted Dry Methane Reforming on Pt-Based Catalysts

**DOI:** 10.1021/acs.energyfuels.6c03688

**Published:** 2026-09-12

**Authors:** James L. Trettin, Janice M. Kankolongo, Cole W. Hullfish, Bruce E. Koel, Michele L. Sarazen

**Affiliations:** † Department of Chemical and Biological Engineering, 6740 Princeton University, Princeton, New Jersey 08540, United States; ‡ Princeton Plasma Physics Laboratory, Princeton, New Jersey 08540, United States

## Abstract

Nonthermal plasma (NTP)-assisted dry methane reforming (DMR) on supported metal catalysts has emerged as a promising method for sustainable, electrified conversion of methane (CH_4_) and carbon dioxide (CO_2_) into carbon monoxide (CO) and hydrogen (H_2_), which are valuable chemical/fuel precursors and energy carriers. NTP-assisted DMR has been reported to enhance reaction rates and reduce catalyst deactivation relative to purely thermal operation. However, the complex interactions between the plasma, metal nanoparticles, and catalyst support under reaction conditions complicate mechanistic interpretation and limit informed catalyst design. Here, plasma-assisted DMR on platinum (Pt) nanoparticles supported on alumina (Al_2_O_3_), silica (SiO_2_), and zeolite 3A was investigated in a coaxial packed-bed dielectric barrier discharge (DBD) reactor to increase understanding of how the plasma, metal, and support interact to govern activity, selectivity, and catalyst stability. The plasma enhanced DMR turnover rates, altered selectivity among DMR, reverse water–gas shift (RWGS), and direct coupling of methane (DCM) pathways as indicated by CO, H_2_, water (H_2_O), and ethane (C_2_H_6_) formation rates, and reduced the apparent activation barriers. The extents of all these plasma-induced changes were impacted by the catalyst identity. Properties of the support (e.g., basic site density and dielectric constant) affected the plasma power and catalytic performance, and the Pt nanoparticle size impacted the plasma and product selectivity. Post-reaction characterization and extracted deactivation rate constants revealed support-dependent catalyst stability, including coke formation and Pt restructuring, with minimal sintering on Al_2_O_3_, significant agglomeration on 3A after plasma exposure, and reduced deactivation of Pt/SiO_2_ under plasma-assisted conditions relative to thermal conditions. These results provide insight into the interplay of plasma, metal nanoparticles, and support in NTP-assisted DMR, with implications for informed catalyst design and process optimization in similar plasma-assisted catalytic C_1_ upgrading systems.

## Graphical abstract

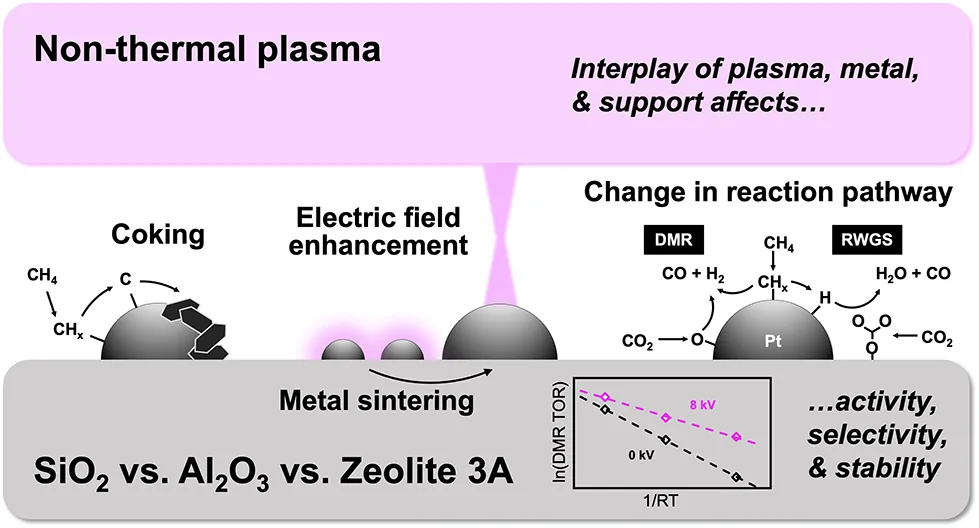

## 1 Introduction

Converting methane (CH_4_) and carbon dioxide (CO_2_) into synthesis gas (syngas; hydrogen (H_2_) + carbon monoxide (CO)) via dry methane reforming (DMR; CH_4_ + CO_2_→2CO + 2H_2_) is an attractive strategy for simultaneous utilization of two primary anthropogenic greenhouse gases and production of value-added fuels and chemicals. However, conventional thermocatalytic DMR is severely limited by its strong endothermicity and high activation barriers for dissociating CH_4_ and CO_2_. These necessitate exceedingly high operating temperatures (>1400 K)^1^ that impose prohibitive energy demands and accelerate catalyst deactivation via coking and metal sintering, that, together, have hindered its widespread industrial implementation. Electrification of DMR via nonthermal plasma (NTP)-assisted DMR has emerged as a promising alternative, in which energetic electrons activate CH_4_ and CO_2_ to generate reactive radicals and vibrationally/electronically excited species at near-ambient thermal conditions.^2,3^ This non-equilibrium activation can increase reaction rates, improve selectivity to desired (and more valuable) products (e.g., oxygenates and C_2+_ hydrocarbons), and enhance catalyst stability, which collectively lower the energy demand for syngas production and enable distributed operation suited to stranded CH_4_ resources.^4^ However, mechanisms of NTP-assisted DMR remain inadequately understood, largely due to complex plasma/catalyst interactions under reaction conditions that may include: (i) plasma-induced modifications of the catalyst, such as the metal oxidation state, particle size, and coordination environment; (ii) catalyst-induced modifications of the plasma, such as electric field distortion and plasma mode switching; and (iii) emergent plasma-catalyst synergies, such as active site density enhancement, coke suppression, and new surface-mediated reaction pathways.^5^ These complex interactions obfuscate mechanistic evaluation and thus complicate rational catalyst design and operating parameter optimization.

Several important contributions have advanced understanding of NTP-assisted DMR. Support properties, particularly dielectric constant,^6,7^ influence discharge behavior and plasma/catalyst coupling for optimized DMR operation.^2,8^ The support dielectric constant influences the electric field distribution and plasma mode,^7^ but its reported effect on catalytic activity remains unclear. While some studies observed enhanced CH_4_ conversion and energy efficiency with higher dielectric constant,^9,10^ others observed the opposite trend,^11,12^ which may be related to changes in the plasma mode or spatial uniformity.^13,14^ Support surface properties, including basic site density/strength^15^ and metal-support interactions (MSI), influence CO_2_ activation, coke formation, and competitive reverse water–gas shift (RWGS; CO_2_ + H_2_→CO + H_2_O) reactions under thermal conditions,^15,16^ but their role in the presence of plasma remains unclear. The identity and size of supported metal nanoparticles further influence activity and selectivity and complicate mechanistic evaluation. Although the optimal metal choice may differ between NTP-assisted and thermal catalytic systems,^17^ plasma-assisted DMR literature has largely explored conventional metals from thermal DMR, such as Ni,^18–21^ Ru, or Pt.^22,23^ Metal nanoparticles can enhance^24,25^ or suppress^26,27^ NTP-assisted DMR conversion and change product selectivities relative to the catalyst support without metal,^28^ but these trends are complicated by nanoparticle (NP) size and morphology effects on plasma/catalyst synergies. It has remained particularly difficult to determine whether catalytic trends originate from changes in bulk plasma properties, local plasma/catalyst interactions, or intrinsic catalytic descriptors that persist under plasma conditions.

Herein, we address these open questions by systematically investigating the interplay of plasma, Pt nanoparticles, and dielectric support in NTP-assisted DMR on 1 wt % Pt supported on γ-Al_2_O_3_, SiO_2_, and zeolite 3A in a coaxial packed-bed dielectric barrier discharge (DBD) reactor. Pt was selected as a model metal because its relatively high coking resistance and structural stability compared with other metals commonly used for DMR (e.g., Ni) facilitate investigation of plasma/catalyst interactions while lessening contributions from catalyst deactivation. γ-Al_2_O_3_, SiO_2_, and zeolite 3A were chosen as catalyst supports to probe the effect of their surface (e.g., basic site density) and dielectric properties on activity, selectivity, and stability, while their narrow range of dielectric constants allows investigating the effect of subtle dielectric changes on the discharge behavior. Kinetic and stability analyses were combined with charge-voltage (*Q*–*V*) characterization of the plasma to evaluate the interface of plasma, metal nanoparticles, and dielectric support. We found that the plasma enhanced DMR turnover rates, altered selectivity as indicated by CO, H_2_, ethane (C_2_H_6_), and H_2_O formation rates, and reduced apparent activation barriers by ∼40 kJ/mol across all catalysts, consistent with enhanced CH_4_ and CO_2_ activation in the plasma. In contrast, RWGS kinetics were largely unchanged, suggesting preferential enhancement of CH_4_ activation in the plasma. Although the reduced electric field (*E*/*N*) of the bulk plasma was mostly insensitive to support identity and Pt incorporation, experiments showed that reactivity, selectivity, and stability varied substantially across catalysts, implicating microdischarges, local electric fields, and plasma/catalyst coupling as influential variables in NTP-assisted DMR. The support dielectric properties exhibited a complex influence on activity, in which a higher dielectric constant did not necessarily correspond to better performance. Further, Pt nanoparticle size effects on the plasma/catalyst coupling were probed on the SiO_2_ support; it was observed that larger nanoparticles (10 nm) enhanced plasma power relative to smaller nanoparticles (2 nm), likely through competing mechanisms of local field enhancement and interfacial polarization. Finally, catalyst stability was assessed by extracting deactivation rate constants, probing extended reaction times, and conducting post-reaction characterization under plasma-assisted and thermal conditions. Plasma exposure mitigated deactivation during DMR, while post-reaction characterization revealed support-dependent coke accumulation and Pt restructuring. Together, these results provide mechanistic guidance for informed catalyst and reactor design in plasma-assisted DMR and related plasma-assisted catalytic C_1_ upgrading systems.

## 2 Materials and Methods

### 2.1 Catalyst Synthesis

1 wt % Pt/γ-Al_2_O_3_, 1 wt % Pt/SiO_2_, and 1 wt % Pt/3A catalysts were prepared via incipient wetness impregnation. Tetraamineplatinum­(II) nitrate (Pt­(NH_3_)_4_(NO_3_)_2_, 99.995%; Sigma-Aldrich) was dissolved in deionized water equal in volume to the pore volume of each support. Beads were chosen to minimize reactor pressure drop and improve plasma homogeneity. γ-Al_2_O_3_ (0.9 mm average bead diameter; Saint-Gobain NorPro), SiO_2_ (1.5 mm average bead diameter; Saint-Gobain NorPro), and zeolite 3A (0.6 mm average bead diameter; Thermo Fisher) were pre-treated under static air at 873 K (2 K min^–1^ ramp) for 2 h. The precursor solution was added dropwise to the beads, followed by drying in static air at 353 K overnight (2 K min^–1^ ramp). This was followed by treatment at 673 K for 3 h under dry air flow (2 K min^–1^ ramp, 50 sccm/g; Airgas, Ultra zero grade) in a tube furnace, reduction at 673 K for 2 h under H_2_ flow (50 sccm/g; Airgas, UHP), and cooling to room temperature under H_2_ flow. The large nanoparticle Pt/SiO_2_ catalyst was synthesized with a secondary treatment at 1023 K for 11 h under dry air flow (2 K min^–1^ ramp, 50 sccm/g), reduction at 673 K for 4 h under H_2_ flow (50 sccm/g), and cooling to room temperature under H_2_ flow.

### 2.2 Nitrogen (N_2_) Physisorption

N_2_ adsorption-desorption isotherms were collected at 77 K with a Micromeritics 3Flex instrument and used to determine multipoint Brunauer-Emmett-Teller (BET) surface areas, density functional theory (DFT) pore volumes, and Barrett-Joyner-Halenda (BJH) pore size distributions (PSD) of the supports. Prior to analysis, samples were degassed at 383 K under mild vacuum (∼125 Torr) overnight. BET surface areas were calculated using BET Surface Identification software in accordance with the Rouquerol criteria.^29^ The total pore volume was determined from the cumulative DFT pore volume up to a pore width of 45 nm, obtained from the DFT PSD. The DFT PSD was generated using a N_2_ Tarazona nonlocal DFT model^30^ (*E*_sf_ = 30.0 K) assuming cylindrical pore geometry and incorporating adsorption data across the full relative pressure range.

### 2.3 CO_2_ Temperature-Programmed Desorption (TPD)

CO_2_ adsorption capacities and characteristics of the supports were determined ex situ with a Micromeritics Autochem II 2920. Approximately 100 mg of support was loaded into a quartz U-tube reactor and pretreated under 30 sccm He flow at 673 K for 1 h. After cooling to 308 K under He, equilibrium CO_2_ adsorption was established over 1 h at 308 K with 30 sccm 10% CO_2_/He flow. The support was then purged with 30 sccm He at 308 K for 30 min to remove weakly bound CO_2_, followed by a temperature ramp to 973 K (10 K min^–1^) with a He flow to monitor desorption via a thermal conductivity detector (TCD).

### 2.4 Scanning/Transmission Electron Microscopy (S/TEM)

Supported Pt nanoparticles were imaged before and after plasma-assisted DMR using a Talos F200X S/TEM operated at 200 keV. Catalyst beads were ground to powder, sonicated 20 min in acetone (>99.9%, Sigma-Aldrich), and deposited onto Formvar carbon-coated 400 mesh Cu grids. Pt nanoparticle size distributions were measured using ImageJ software from >200 particles per sample.

### 2.5 Thermogravimetric Analysis (TGA)

Carbon deposition on spent catalysts was quantified with a PerkinElmer TGA 8000. Samples were first heated and held at 383 K under Ar to remove water, followed by a ramp to 1173 K (10 K min^–1^) under 50 sccm dry air. The mass loss profile was recorded to monitor carbon deposition and coke content, and derivative thermogravimetric (DTG) curves were filtered using a 101-point Savitzky-Golay filter.

### 2.6 CO-Pulse Chemisorption

Pt dispersions were measured before and after plasma-assisted DMR with a Micromeritics Autochem II 2920. Approximately 100 mg of catalyst was reduced in situ under 10% H_2_/Ar flow (50 sccm; Airgas) at 673 K (10 K min^–1^ ramp) for 60 min. Samples were purged under He (50 sccm; Airgas, UHP) at 673 K for 30 min, then cooled to 308 K and held for 10 min to stabilize the TCD. Injections of 10% CO (balance He) were introduced every 6 min until saturation was observed. Pt dispersion was calculated by the following equation:

$$\mathrm{Dispersion}=\frac{n_{\mathrm{CO}}}{n_{\mathrm{Pt}}}\times F$$ 1

where *n*_CO_ is the moles of chemisorbed CO, *n*_Pt_ is the moles of Pt in the sample, and *F* is the stoichiometric factor, which was assumed to be 1. Fractions of Pt surface sites existing as Pt step, edge, and corner sites (i.e., under-coordinated, UC sites) and as Pt terraces (i.e., well-coordinated, WC sites) were estimated using the dispersion measured by CO-pulse chemisorption and assuming a cubo-octahedral nanoparticle geometry calculated via a Wulff construction.^31,32^

### 2.7 CO Diffuse Reflectance Infrared Fourier Transform Spectroscopy (DRIFTS)

Pt nanoparticle size and oxidation state were probed with a Bruker INVENIO FTIR with a liquid N_2_ cooled MCT detector and a diffuse-reflection high-temperature reaction chamber (Praying Mantis, Harrick Scientific) with ZnSe windows. Undiluted samples (∼40 mg) were pretreated in situ at 15 Torr under 100 sccm 10% H_2_/Ar at 673 K for 1 h (10 K min^–1^ ramp), flushed with 50 sccm Ar for 30 min, and cooled to 303 K. CO adsorption was performed with 100 sccm 10% CO/He for 10 min, followed by a 10 min Ar purge. DRIFTS spectra were collected from 4000 to 600 cm^–1^ at 4 cm^–1^ resolution and averaged over 10 scans.

### 2.8 Plasma Characterization

Charge-voltage (*Q*–*V*) characteristics, i.e., Lissajous figures, were recorded using a high voltage probe (1000× attenuation; P6015, Tektronix) and a four-channel digital storage oscilloscope (GDS-1054B, GW Instek). The voltage (*V*) across the reactor is related to the accumulated charge (*Q*) and the effective capacitance (*C*_eff_), which includes contributions from the dielectric barrier, gas gap, and packed bed structure according to

2 $$V=\frac{Q}{C_{\mathrm{eff}}}$$

Plasma power was calculated by numerical integration of the Lissajous figure as below:

3 P=f∮V(t)dQ(t)

where *f* is the AC frequency (20 kHz). The slope of the *Q*–*V* plot was evaluated to qualitatively assess trends in the reduced electric field (*E*/*N*) of the bulk plasma. The dielectric capacitance of the cell, *C*_cell_, is proportional to the slope of the *Q*–*V* plot, and the burning voltage, *U*_b_, is inversely related to *C*_cell_ as follows:^33–35^

4 $$U_{b}=\frac{1}{1+\frac{C_{\mathrm{cell}}}{C_{d}-C_{\mathrm{cell}}}}U_{\min}$$

where *C*_d_ is the capacitance of the dielectric barrier and *U*_min_ is the minimum voltage needed to sustain the discharge. *E*/*N* is directly proportional to the burning voltage:

$$E/N=\frac{U_{b}}{g\times N}$$ 5

where *g* is the discharge gap width and *N* is the total number density of the gas, with E/N expressed in Td. Therefore, an increase in the slope of the *Q*–*V* plot was qualitatively correlated with a decrease in the *E*/*N* value of the bulk plasma.

### 2.9 Plasma-Assisted DMR in a Packed-Bed DBD Reactor

Reactions were performed in a vertically oriented coaxial packed-bed DBD reactor, with a 3/4 in. bed of catalyst beads in the annular region between inner and outer quartz tubes (Figure 1). The inner quartz tube housed a stainless-steel electrode, and an outer copper mesh electrode was wrapped around the outer quartz tube. Plasma in the annular gap where the catalyst bed was situated was generated by a high-frequency (20 kHz), high voltage AC power supply (PVM/DDR Plasma Driver, GME PM89 Class 2.5) and most reactions were performed at an applied voltage of 8 kV. Photographs of the packed-bed DBD reactor are provided in Figure S1, which illustrate the catalyst bed location, direction of gas flow, electrode configuration, and plasma discharge region.

**Figure 1:**
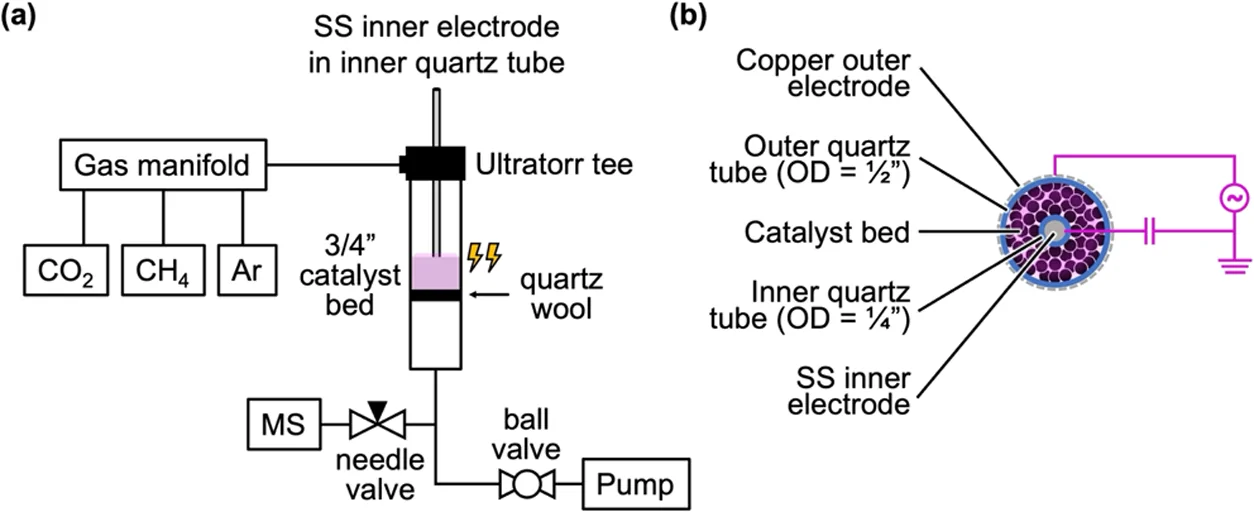
(a) Flow and (b) electrical and packing schematics of the coaxial packed-bed DBD reactor for plasma-assisted DMR experiments.

Catalysts were pretreated in situ under 100 sccm 20% H_2_/Ar at 673 K for 1 h and cooled under Ar. Reactants (40 sccm CH_4_, 40 sccm CO_2_, and 20 sccm Ar) were introduced at 15 Torr. The reaction temperature was controlled up to ∼773 K in a furnace with a thermocouple for feedback control and a secondary thermocouple on the exterior of the reactor near the discharge zone for independent temperature measurement and calibration. Product formation rates were measured by online mass spectrometry (RGA-100, Stanford Research Systems) and normalized by Pt surface sites measured from CO-pulse chemisorption to calculate turnover rates (TOR):

$${TOR}_{i}=\frac{{ṅ}_{i}}{\mathrm{mass}\;\%\;\mathrm{Pt}\times m_{\mathrm{cat}}\times \mathrm{dispersion}\times \frac{1}{M_{\mathrm{Pt}}}}\left[=\right]\mathrm{mol}\;i\cdot \mathrm{mol}{\;\mathrm{Pt}}^{-1}\cdot s^{-1}$$ 6

where *ṅ* **_i_** is the molar flow rate of species *i* $\left[\frac{\mathrm{mol}\;i}{s}\right]$ , the mass % Pt was 1%, *m*_cat_ is the mass of catalyst, and *M*_Pt_ is the atomic mass of Pt (195.1 g/mol). Steady-state reactant and product turnover rates were typically achieved within 3 min at a given condition. In cases of measurable deactivation in the rate, a pseudo-first-order deactivation rate constant was extracted by fitting the natural logarithm of the CO, H_2_, and H_2_O turnover rates to a linear function of time:

7 $${TOR}_{i}={TOR}_{i,0}e^{-k_{d}t}$$

8 $$\ln\left({TOR}_{i}\right)=\ln\left({TOR}_{i,0}\right)-k_{d}t$$

where TOR*_i_* is the instantaneous turnover rate of product *i*, TOR_*i*,0_ is the initial turnover rate of product *i*, and *k*_d_ is the pseudo-first-order deactivation rate constant. Product turnover rates were used for this analysis to directly track changes in product formation with time on stream, although deactivation may equivalently be assessed from reactant consumption rates. The extracted *k*_d_ values were used to correct initial turnover rates used for kinetic analyses. Apparent activation barriers were determined from Arrhenius plots over the temperature range of 573–696 K for conversions ≤ 5%, with errors reported as ± 2σ from linear regression analysis. Fractional H_2_, CO, H_2_O, and ethane (C_2_H_6_) product selectivities (*S*_i_) were calculated as

9 $$S_{i}=\frac{{ṅ}_{i}}{\sum_{i}{ṅ}_{i}}$$

where ∑*_i_ṅ_i_* represents the sum of all product formation rates. H_2_/CO product ratios were calculated from measured H_2_ and CO formation rates and are only reported at conditions above the reliable quantification limit. Carbon balances were calculated from the measured flow rates of all carbon-containing species as

$$C\;balance\;\left(\%\right)=\frac{{ṅ}_{{\mathrm{CH}}_{4}}+{ṅ}_{{\mathrm{CO}}_{2}}+{ṅ}_{\mathrm{CO}}+2\times {ṅ}_{C_{2}H_{6}}}{{ṅ}_{{\mathrm{CH}}_{4},\mathrm{in}}+{ṅ}_{{\mathrm{CO}}_{2},\mathrm{in}}}\times 100$$ 10

The extent of reaction for DMR, RWGS, and DCM (ξ_i_) was calculated as

$$\xi_{\mathrm{RWGS}}={ṅ}_{H_{2}O};\xi_{\mathrm{DCM}}=2\times {ṅ}_{C_{2}H_{6}};\xi_{\mathrm{DMR}}=\frac{{ṅ}_{\mathrm{CO}}-\xi_{\mathrm{RWGS}}}{2}$$ 11

CH_4_ selectivities to DMR and DCM were defined as fractional conversions of CH_4_ via DMR and DCM pathways, respectively, as

12 $$S_{{\mathrm{CH}}_{4},\mathrm{DMR}}=\frac{\xi_{\mathrm{DMR}}}{\xi_{\mathrm{DMR}}+\xi_{\mathrm{DCM}}};S_{{\mathrm{CH}}_{4},\mathrm{DCM}}=\frac{\xi_{\mathrm{DCM}}}{\xi_{\mathrm{DMR}}+\xi_{\mathrm{DCM}}}$$

and CO_2_ selectivities to DMR and RWGS were defined as fractional conversions of CO_2_ via DMR and RWGS pathways, respectively, as

13 $$S_{{\mathrm{CO}}_{2},\mathrm{DMR}}=\frac{\xi_{\mathrm{DMR}}}{\xi_{\mathrm{DMR}}+\xi_{\mathrm{RWGS}}};S_{{\mathrm{CO}}_{2},\mathrm{RWGS}}=\frac{\xi_{\mathrm{RWGS}}}{\xi_{\mathrm{DMR}}+\xi_{\mathrm{RWGS}}}$$

Thermodynamic equilibrium conversions for DMR were calculated from the standard reaction enthalpy and entropy values obtained from the NIST Chemistry WebBook.^36^ The standard Gibbs free energy of reaction at each temperature was calculated as

14 $${\Delta G}_{rxn}^{0}\left(T\right)={\Delta H}_{rxn}^{0}-T{\Delta S}_{rxn}^{0}$$

from which the equilibrium constant was obtained according to

15 $$K=e^{\left(-{\Delta G}_{rxn}^{0}/\mathrm{RT}\right)}$$

Equilibrium CH_4_ and CO_2_ conversions were then determined from the DMR stoichiometry and the experimental feed composition (40% CH_4_, 40% CO_2_, 20% Ar) and total pressure (15 Torr).

## 3 Results and Discussion

### 3.1 Thermal DMR on Supported Pt Catalysts

Before investigation of plasma-assisted DMR, thermal rates and selectivities were assessed on the catalyst samples. Catalyst samples synthesized targeting 2–3 nm nanoparticles on the varied oxide supports were characterized using CO-pulse chemisorption and TEM. Pt nanoparticle sizes measured by TEM were similar for Al_2_O_3_ and SiO_2_ (∼2 nm) and slightly larger on 3A (∼3.5 nm), consistent with Pt dispersions of 0.59, 0.40, and 0.27 measured via CO-pulse chemisorption for fresh 1 wt % Pt/Al_2_O_3_, Pt/SiO_2_, and Pt/3A, respectively (Figure S2; Table 1). The slightly larger nanoparticles on 3A are consistent with its small pores inaccessible to the Pt precursors such that all Pt is relegated to the external surface of the zeolite crystals. This is reflected by higher BET surface areas from N_2_ physisorption for Al_2_O_3_ and SiO_2_ compared to 3A (Figure S3, Table 1), as the 0.3 nm micropores of 3A are inaccessible to both the Pt precursor during synthesis and to N_2_ during physisorption measurements.

**Table 1:** BET Surface Areas and DFT Pore Volumes, Pt Dispersions with Calculated Under-Coordinated (UC) Pt Fractions via Wulff Construction, and CO_2_ Uptake Quantities for Supported Pt Catalysts from N_2_ Physisorption, CO-Pulse Chemisorption and TEM, and CO_2_ TPD, Respectively

1 wt % Pt catalyst	BET area^a^ [m^2^ g^–1^]	Pore volume^a^ [cm^3^ g^–1^]	Pt dispersion	*d*_NP,CO_ [nm]	*d*_NP,TEM_ [nm]	UC^b^ Pt fraction	CO_2_ capacity^c^ [mmol-CO_2_ g^–1^]
Pt/Al_2_O_3_	280	0.75	0.59	1.9	1.8 ± 0.6	0.60	0.20
Pt/SiO_2_ (2 nm)	265	0.29	0.40	2.8	1.9 ± 1.1	0.37	0.04
Pt/SiO_2_ (10 nm)	265	0.29	0.13	10.5	10.4 ± 5.4	0.11	0.04
Pt/3A	30	0.07	0.27	4.2	3.5 ± 4.5	0.24	0.36

a Measured from N_2_ physisorption for metal-free supports.

b UC = under-coordinated (i.e., steps, edges, and corners).

c Measured from CO_2_ TPD for metal-free supports.

Steady-state thermal CO, H_2_, H_2_O, and C_2_H_6_ turnover rates, normalized by Pt dispersions via CO-pulse chemisorption, were measured on each supported Pt catalyst from 473 to 748 K (P_CH4_ = 6 Torr, P_CO2_ = 6 Torr, P_Ar_ = 3 Torr; 100 sccm total flow) in the packed-bed reactor under near differential conversion regimes (< 15%) (Figure 2), with no measurable thermal conversion on metal-free supports. Low conversions were intentionally maintained to facilitate kinetic analysis. Calculated DMR equilibrium conversions were substantially higher than the experimentally measured conversions over the investigated conditions (Figure S6), with measured conversions remaining below 30% of the corresponding equilibrium conversion under all conditions. CO and H_2_ turnover rates increased exponentially above 550 K on all catalysts, while the onset of H_2_O and C_2_H_6_ turnovers was observed at higher temperatures of 600 and 650 K, respectively. H_2_O formation (Figure 2c) and H_2_/CO product ratios <1 (Figure S15a) on Pt/Al_2_O_3_ and Pt/3A indicate competitive reverse water–gas shift (RWGS), which consumes H_2_ and produces additional CO and H_2_O. Although the H_2_/CO ratios increased with temperature for these catalysts, they remained <1 at even the highest temperature, reaching 0.80 on Pt/Al_2_O_3_ and 0.33 on Pt/3A. In contrast, the H_2_/CO ratio on Pt/SiO_2_ was near 1 at all temperatures, consistent with negligible H_2_O formation and minimal RWGS activity. Carbon balances remained above 99% for all supported Pt catalysts at all conditions tested (Figure S6c).

**Figure 2:**
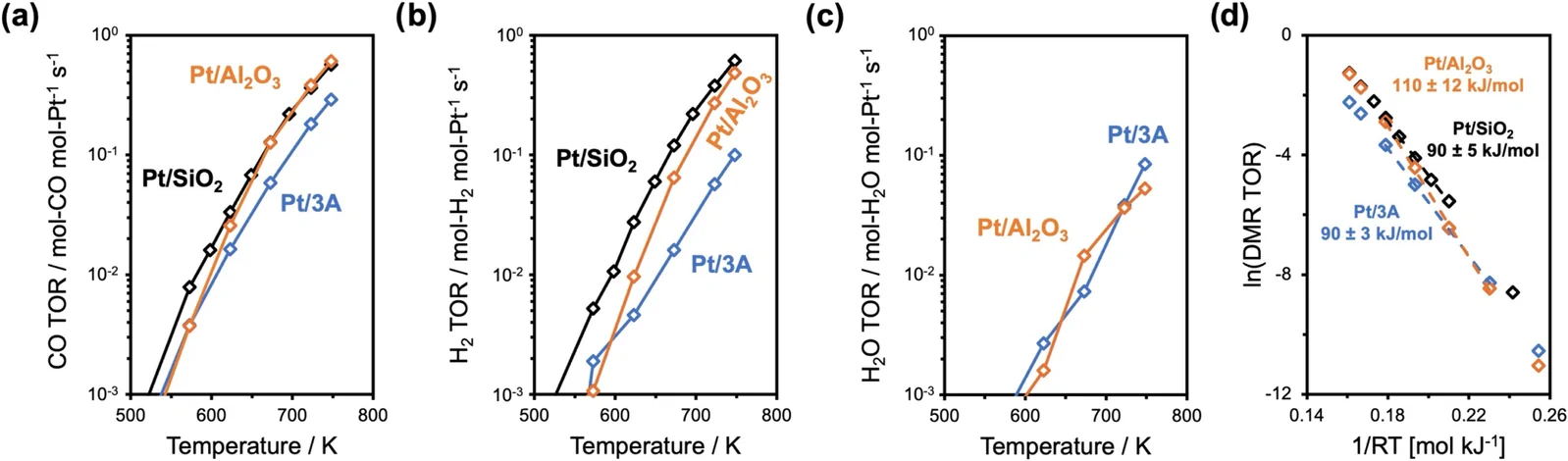
Steady-state thermal turnover rates for (a) CO, (b) H_2_, and (c) H_2_O, and (d) Arrhenius plots for thermal DMR on Pt/Al_2_O_3_ (orange), Pt/SiO_2_ (black), and Pt/3A (blue). Reaction conditions: P_CH4_ = P_CO2_ = 6 Torr, P_Ar_ = 3 Torr, and 100 sccm total flow.

Deactivation was observed on Pt/SiO_2_, as evidenced by slowly decreasing product turnover rates over time at constant temperature (Figure S5a). At each temperature, apparent pseudo-first-order deactivation rate constants (*k*_d_) were extracted for CO and H_2_ over a 5 min isothermal period to assess the temperature dependence of deactivation; a representative fit at 698 K is provided in Figure S5c, and the resulting values are summarized in Figure S5d. While apparent deactivation rate constants for CO steadily increased from 0.5 to 1.1 h^–1^ as the temperature increased from 598 to 748 K, the deactivation rate constants for H_2_ remained nearly constant, increasing only from 1.0 to 1.1 h^–1^ over the same temperature range (Figure S5d). The stronger temperature dependence of CO deactivation suggests a thermally activated deactivation process for sites associated with CO formation (i.e., Pt terrace or interfacial sites involved in CO_2_ activation).^37–39^ In contrast, the weak temperature dependence of H_2_ deactivation suggests that sites associated with H_2_ formation (i.e., Pt step and edge sites involved in CH_4_ activation)^40–42^ appear less sensitive to temperature over this range. Site-dependent and temperature-dependent deactivation via coking or surface restructuring during DMR has been reported on similar supported metal catalysts.^43,44^

To distinguish sustained catalyst deactivation from short-time scale rate transients at various temperatures, global deactivation constants (denoted *k*_d_^*’*^, 0.82 h^–1^ for CO and 0.88 h^–1^ for H_2_) were determined from turnover rates measured at 623 K before and after the temperature ramp and applied to correct rates across the full temperature range. Less deactivation was observed on Pt/3A (Figure S5b), with global deactivation rate constants of 0.29 h^–1^ (CO), 0.31 h^–1^ (H_2_), and 0.34 h^–1^ (H_2_O) applied to the turnover rates, and no measurable deactivation was observed on Pt/Al_2_O_3_. Significant deactivation on Pt/SiO_2_ relative to Pt/3A and Pt/Al_2_O_3_ is attributed to enhanced coke formation consistent with a lack of metal/support interfacial sites that stabilize CO_2_-derived oxygen-containing intermediates for the inert SiO_2_ support,^45^ as will be discussed later (Section 3.4). All thermal DMR and RWGS reaction rates and selectivities that follow have been adjusted for deactivation accordingly.

CO turnover rates were highest on Pt/SiO_2_ below 700 K and highest on Pt/Al_2_O_3_ above this temperature, while Pt/3A exhibited the lowest CO turnover rates at all temperatures (Figure 2a). Across the entire temperature range, H_2_ turnover rates followed the trend Pt/SiO_2_ > Pt/Al_2_O_3_ > Pt/3A. Higher CO and H_2_ turnover rates on Pt/Al_2_O_3_ and Pt/SiO_2_ relative to Pt/3A are consistent with the reported structure-sensitivity of CH_4_ adsorption and dissociation on Pt, which has been identified as the rate-determining step in other studies.^45^ Low-coordination Pt edge and corner sites bind CH_*x*_ intermediates more strongly and lower the CH_4_ activation energy.^40^ The higher fraction of low-coordination Pt step, edge, and corner sites on Al_2_O_3_ (0.60) and SiO_2_ (0.37) relative to 3A (0.24) increases the density of sites that facilitate CH_4_ dissociation, contributing to higher CO and H_2_ turnover rates (Table 1).

The near-total CH_4_ selectivity to DMR for all catalysts at all temperatures indicates that CH_4_ primarily reacts via DMR, with minimal contribution from the direct coupling of methane (DCM; 2CH_4_→H_2_ + C_2_H_6_) (Figure S7b). This is corroborated by the low C_2_H_6_ turnover rates (< 0.01 mol-C_2_H_6_ mol-Pt^–1^ s^–1^) across the full temperature range on all catalysts (Figure S7a). C_2_H_6_ turnover rates were higher on Pt/Al_2_O_3_ (0.0082 mol-C_2_H_6_ mol-Pt^–1^ s^–1^ at 748 K) relative to Pt/SiO_2_ and Pt/3A (0.0018 and 0.0019 mol-C_2_H_6_ mol-Pt^–1^ s^–1^ at 748 K, respectively), likely related to higher fractions of Pt step and edge sites (0.60) on the smaller nanoparticles for Pt/Al_2_O_3_ that are more active for DCM.^46^ Apparent activation barriers for thermal DMR, calculated from net DMR rates derived from eq 11 between 523 and 673 K (Figure 2d), were 90 ± 5, 90 ± 3, and 110 ± 12 kJ/mol for Pt/SiO_2_, Pt/3A, and Pt/Al_2_O_3_, respectively, in agreement with values reported in literature for non-reducible metal oxide-supported Pt catalysts (Figure 2d).^47,48^ The slightly higher apparent barrier for Pt/Al_2_O_3_ could reflect differences in relative surface coverages of adsorbed CH_x,_ CO, or oxygen-containing intermediates.^20,21^

CO_2_ selectivities to DMR, however, were below 100% (Figure S7c) and measurable H_2_O turnover rates for Pt/Al_2_O_3_ and Pt/3A (Figure 2c) indicate competitive RWGS on those catalysts. For example, CO_2_ selectivities to DMR at 748 K were 55%, 84%, and 100% for Pt/3A, Pt/Al_2_O_3_, and Pt/SiO_2_, respectively. Consistent with these selectivity trends, H_2_/CO product ratios increased with temperature but remained slightly <1 (the stoichiometric value for DMR) for Pt/Al_2_O_3_ (0.80 at 748 K) and far below 1 for Pt/3A (0.33 at 748 K). This trend is consistent with the higher CO_2_ adsorption capacities measured for 3A (0.36 mmol CO_2_ g^–1^) and Al_2_O_3_ (0.20 mmol CO_2_ g^–1^) relative to SiO_2_ (0.04 mmol CO_2_ g^–1^) (Figure S4, Table 1). The basic sites on 3A (nominally associated with K^+^-exchanged sites)^49^ and Al_2_O_3_ (O^2–^ or OH groups)^50^ may stabilize carbonate/bicarbonate intermediates relevant to RWGS. Previous studies on similar oxide-supported metal nanoparticle catalysts have proposed that these intermediates participate in RWGS through hydrogen spillover (H-spillover) from Pt to the metal/support interface, where they are subsequently hydrogenated to CO.^51,52^ Apparent activation barriers for RWGS based on H_2_O turnover rates were measured to be 107 ± 20 kJ/mol and 105 ± 16 kJ/mol on Pt/Al_2_O_3_ and Pt/3A, respectively (Figure S8); these apparent barriers reflect in situ H_2_ concentration as produced by DMR, adsorption equilibria, and intrinsic RWGS kinetics. While RWGS rates could be expected to increase with greater metal/support interfacial area for the smaller Pt nanoparticles of Pt/Al_2_O_3_, the similar measured RWGS rates on Pt/Al_2_O_3_ and Pt/3A suggest that the greater density of basic sites on Pt/3A compensates for the larger Pt nanoparticle size.^53^

Since DMR is thermodynamically favored over RWGS at elevated temperatures due to its higher endothermicity (ΔH ∼+247 kJ/mol for DMR and +41 kJ/mol for RWGS),^54^ the observed decrease in CO_2_ selectivity to DMR with increasing temperature on Pt/3A indicates that selectivity is governed by the relative kinetics of competing reactions rather than thermodynamic equilibrium (Figure S7c). This is reflected in the higher apparent activation barrier for RWGS on Pt/3A (105 kJ/mol) relative to DMR (90 kJ/mol) that results in increasingly competitive RWGS at elevated temperatures. While DMR is limited by CH_4_ activation and oxidation of adsorbed [C*] species,^45^ RWGS is primarily limited by H-spillover to carbonate/bicarbonate species on the support,^55,56^ which occurs at metal/support interfacial sites. The high density of basic sites on 3A may increase the concentration of these intermediates, as suggested by the higher CO_2_ adsorption capacity of 3A (Table 1), and higher temperatures increase H_2_ formation from DMR and thus the rate of H-spillover that further promotes RWGS. In contrast, the activation barrier for RWGS on Pt/Al_2_O_3_ (107 ± 20 kJ/mol) was nearly identical to that for DMR (110 ± 12 kJ/mol), consistent with stable CO_2_ selectivities to DMR over the entire temperature range.

### 3.2 Plasma-Assisted DMR on Metal-Free Supports

Steady-state plasma-assisted CO, H_2_, and C_2_H_6_ formation rates (Figure 3) were measured on the metal-free supports from 473 to 723 K (P_CH4_ = 6 Torr, P_CO2_ = 6 Torr, P_Ar_ = 3 Torr; 100 sccm total flow) in the packed-bed reactor, corresponding to differential CH_4_ and CO_2_ conversions (<3%) with carbon balances above 99% at all conditions tested (Figure S9). Conversions, H_2_/CO ratios, H_2_O and C_2_H_6_ formation rates, and carbon balances at 673 K are summarized in Table S1. Unlike the thermal case, measurable DMR activity was observed on the metal-free supports with plasma; however, no H_2_O was formed under any NTP conditions tested here, indicating the necessity of Pt sites for plasma-assisted RWGS and for any thermal DMR-related reactions. Along with the absence of detectable H_2_O formation, H_2_/CO ratios near the stoichiometric DMR value of 1 (1.0 ± 0.2) across all temperatures for all metal-free supports (Figure S15b) indicate that syngas formation occurred primarily through DMR.

**Figure 3:**
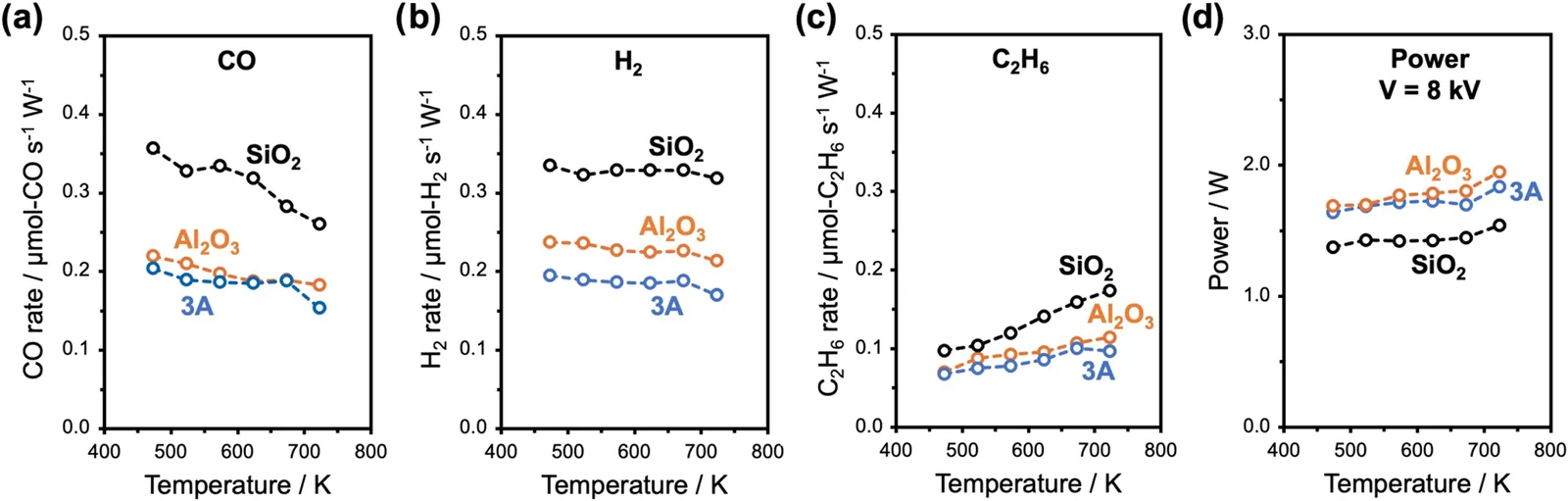
Steady-state power-normalized formation rates for (a) CO, (b) H_2_, and (c) C_2_H_6_, and (d) associated powers extracted from *Q*–*V* analysis for metal-free Al_2_O_3_ (orange), SiO_2_ (black), and 3A (blue). Reaction conditions: P_CH4_ = P_CO2_ = 6 Torr, P_Ar_ = 3 Torr, and 100 sccm total flow with an applied voltage of 8 kV.

Plasma power was extracted via *Q*–*V* analysis (Figure 3d) to normalize product formation rates by power input. Sample *Q*–*V* plots at 473 and 723 K are presented in Figure S10b and S10c, respectively. The measured plasma power slightly increased with temperature for all metal-free supports, likely due to higher gas conductivity at elevated temperatures. Trends in the reduced electric field (*E*/*N*) for the bulk plasma, which can be inferred to scale inversely with the slope of a *Q*–*V* plot as described in the Methods section, did not strongly depend on the packing material. This is evidenced by the qualitatively similar *Q*–*V* plot shapes observed for all metal-free supports, suggesting that differences in activity likely arise from local microdischarge behavior and surface interactions rather than from changes in the bulk electron energy distribution. Plasma power increased with increasing values of the support dielectric constant; power was lowest for SiO_2_ (ε = 4),^57^ intermediate for 3A (ε = 4 – 9),^58^ and highest for Al_2_O_3_ (ε = 9 – 10).^57,59^ Previous studies found that the plasma breakdown voltage decreases as the dielectric constant of the support increases.^60^ A lower breakdown voltage may translate to enhanced electron energy, consistent with higher power, but microfilaments in the plasma may be restricted to bead contact points that lower gas activation by bulk plasma and thus NTP-assisted DMR activity.^14,61^ In contrast, the lower dielectric constant for SiO_2_ may result in more spatially uniform plasma behavior, which is postulated to improve CH_4_ and CO_2_ activation through enhanced formation of reactive species (e.g., radicals, vibrationally excited molecules, and surface intermediates). These trends in plasma power may explain the higher product formation rates for SiO_2_ relative to Al_2_O_3_ and 3A.

While product formation rates varied in magnitude across samples, CO and H_2_ formation rates decreased and C_2_H_6_ formation rates increased with temperature for all metal-free supports. CH_4_ selectivities to DMR decreased linearly with temperature (Figure S11a), suggesting that increasing temperature promotes CH_3_ radical formation and/or recombination to form C_2_H_6_, postulated to proceed through the methane coupling pathway described before.^62–64^ Indeed, several previous studies have identified C_2_H_6_ as the primary product of CH_4_ decomposition in NTP.^3^ CH_4_ selectivities to DMR were slightly higher for SiO_2_ compared to 3A and Al_2_O_3_ over most of the temperature range (e.g., 41, 37, and 35% at 573 K, respectively), and plasma power was slightly lower for SiO_2_ compared to 3A and Al_2_O_3_ (1.44 W, 1.72 W, and 1.79 W, respectively, averaged over the entire temperature range). Reduced surface quenching likely increased steady-state densities of gas-phase radicals,^65^ promoting formation rates of both reforming products (CO, H_2_) and DCM products (H_2_, C_2_H_6_). Interestingly, CH_4_ selectivities for DMR decreased with power for all samples (Figure S11b), consistent with increased radical flux at higher plasma power that enhances CH_3_ radical formation and recombination to form C_2_H_6_.^66^

While the AC power source was operated at identical input settings, measured voltages across the bed varied slightly for different packing materials (Figure S10a), which were 7.4 ± 0.3 kV for 3A, 7.7 ± 0.2 kV for SiO_2_, and 8.1 ± 0.2 kV for Al_2_O_3_ averaged over the entire temperature range. Measured voltages slightly decreased (< 1 kV) with increased temperature for all materials, consistent with a lower sustaining voltage for the discharge as temperature increases.^67^ The measured voltage is inversely related to the effective capacitance of the packed bed, which acts as a dielectric in the plasma gap (see Methods). Slightly smaller beads of 3A (*d*_bead_ = 0.6 mm) relative to Al_2_O_3_ and SiO_2_ (*d*_bead_ = 0.9 mm and 1.5 mm, respectively) pack more tightly and decrease the void space, which increases the effective capacitance and slightly lowers the voltage across the bed.^61^ These results demonstrate the effect the support bead packing has on product formation rates and selectivities in NTP-assisted DMR, even for traditional thermal catalyst supports that are considered “catalytically inert”.

### 3.3 Plasma-Assisted DMR on Supported Pt Catalysts

The effects of support bead packing on product formation rates and reaction pathway selectivities provide context for plasma-assisted DMR activity on supported Pt catalysts. Steady-state plasma-assisted CO, H_2_, C_2_H_6_, and H_2_O turnover rates were measured on supported Pt catalysts from 473 to 723 K (P_CH4_ = 6 Torr, P_CO2_ = 6 Torr, P_Ar_ = 3 Torr; 100 sccm total flow) in the packed-bed reactor (Figure 4), corresponding to differential CO_2_ and CH_4_ conversions (< 6%) with carbon balances >99% at all conditions tested (Figure S12). A quantitative comparison of reaction performance at 673 K is provided in Table S1. These turnover rates were normalized by the plasma power extracted from *Q*–*V* plots (Figure S13b), with representative plots presented in Figure S14a and S14b at 473 and 723 K, respectively. Syngas product turnover rates were generally highest for Pt/Al_2_O_3_, followed by Pt/SiO_2_ and Pt/3A, reflecting the combined influence of support properties and Pt nanoparticle size. This trend is consistent with higher activity on smaller Pt nanoparticles, which have more Pt step and edge active sites for CH_4_ activation. H_2_/CO product ratios were near the stoichiometric DMR value of 1 at 473 K (0.87 to 1.07) for all supported Pt catalysts (Figure S15c). While H_2_/CO remained near 1 at all temperatures for Pt/SiO_2_, H_2_/CO decreased with increasing temperature for Pt/3A and Pt/Al_2_O_3_, in contrast to the increasing H_2_/CO trend observed for these catalysts during thermal DMR. Together with measurable H_2_O formation on Pt/3A and Pt/Al_2_O_3_, this trend is consistent with increasing contributions from RWGS at elevated temperatures.

**Figure 4:**
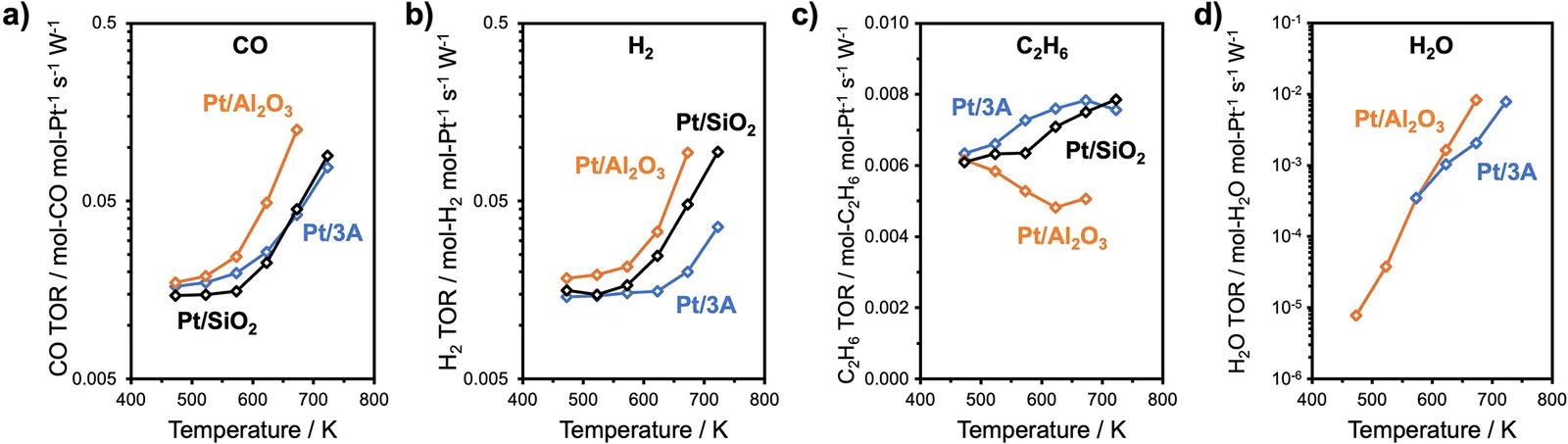
Steady-state power-normalized turnover rates for (a) CO, (b) H_2_, (c) C_2_H_6_, and (d) H_2_O during NTP-assisted DMR on Pt/Al_2_O_3_ (orange), Pt/SiO_2_ (black), and Pt/3A (blue). Reaction conditions: P_CH4_ = P_CO2_ = 6 Torr, P_Ar_ = 3 Torr, and 100 sccm total flow with an applied voltage of 8 kV.

Consistent with the metal-free supports, *E*/*N* for the bulk discharge was largely independent of the identity of the supported Pt catalyst as evidenced by qualitatively similar *Q*–*V* plots. Furthermore, the *Q*–*V* plots for the supported Pt catalysts closely resembled those for the metal-free supports, suggesting that Pt nanoparticles had minimal influence on the bulk electron energy distribution in the plasma. Plasma power increased only marginally with temperature for all supported Pt catalysts and was lowest for Pt/SiO_2_ (Figure S13b), in line with trends observed for the metal-free supports. However, the plasma power was higher for Pt/3A than for Pt/Al_2_O_3_, in contrast to the trend observed for the corresponding metal-free supports. This difference may arise from higher conversions on Pt/Al_2_O_3_ relative to Pt/3A (Figure S12), which would increase gas-phase H_2_ and CO concentrations and potentially influence the discharge. For example, CH_4_ and CO_2_ conversions at 673 K were 2.6 and 1.6% for Pt/3A and 5.2 and 5.1% for Pt/Al_2_O_3_, respectively. However, the weak dependence of the plasma power on temperature suggests that such composition changes are unlikely to dominate this effect.

The effect of Pt nanoparticles on the plasma power was further examined by comparing the plasma power ratio that was obtained by the plasma power for the supported Pt catalyst divided by the plasma power for the respective metal-free support (Figure 5a). The power ratio was slightly above 1.0 for (Pt/)­3A, slightly below 1.0 for (Pt/)­SiO_2_, and farther below 1.0 for (Pt/)­Al_2_O_3_ at all temperatures (1.06 ± 0.02, 0.96 ± 0.04, and 0.89 ± 0.02, respectively, averaged over the entire temperature range). These results indicate enhanced plasma power with Pt on 3A and suppressed plasma power with Pt on SiO_2_ and Al_2_O_3_. The plasma power ratio was independent of temperature for all three materials, suggesting that minor changes in gas composition with increasing conversions likely do not explain this phenomenon. Furthermore, the measured applied voltage ratios (for Pt catalyst/metal-free support) were nearly 1.0 at all temperatures for all catalysts (Figure S16), demonstrating the plasma power ratio is also not influenced by the measured changes in applied voltage.

**Figure 5:**
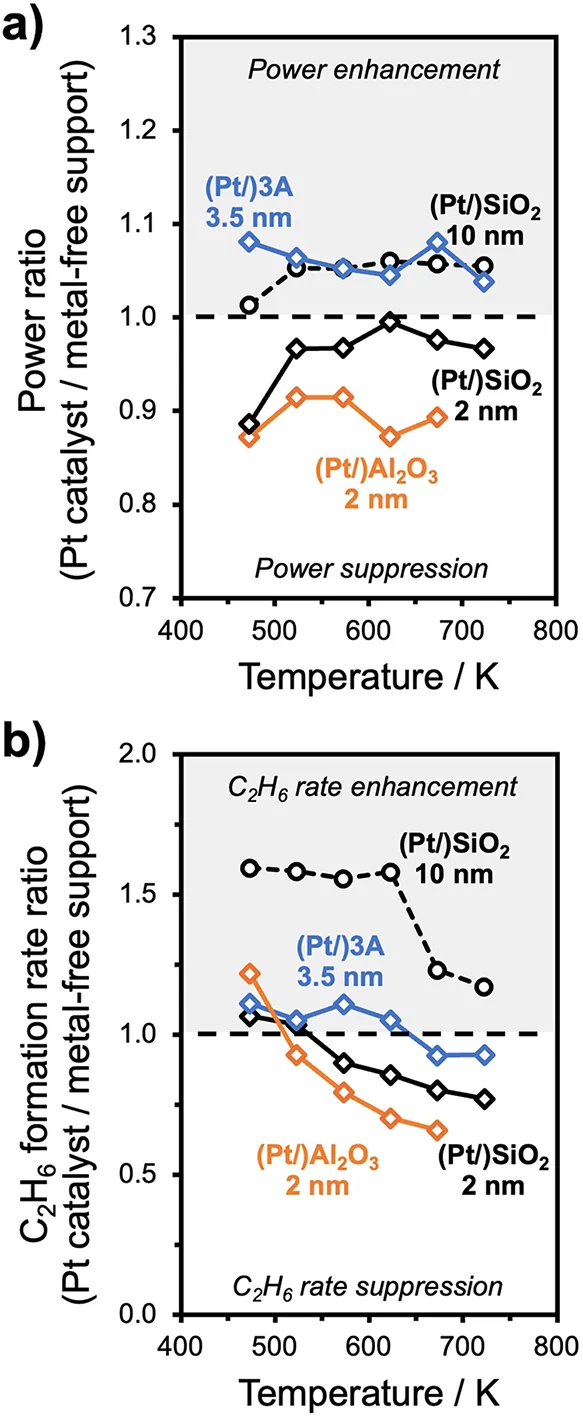
(a) Plasma power ratio and (b) C_2_H_6_ formation rate ratio, defined for the Pt catalyst relative to the metal-free support, during NTP-assisted DMR on (Pt/)­Al_2_O_3_ (2 nm, orange), (Pt/)­SiO_2_ (2 nm, black diamonds, solid line; 10 nm, black circles, dashed line), and (Pt/)­3A (3.5 nm, blue). Reaction conditions: P_CH4_ = P_CO2_ = 6 Torr, P_Ar_ = 3 Torr, and 100 sccm total flow with an applied voltage of 8 kV.

Instead, the plasma power ratio varied systematically with Pt nanoparticle size. The highly dispersed, small Pt nanoparticles on Al_2_O_3_ and SiO_2_ suppressed the plasma power relative to their corresponding metal-free supports, while the larger Pt nanoparticles on 3A enhanced the plasma power. This was further demonstrated by plasma power ratios >1 at all temperatures (1.05 ± 0.02) for a large nanoparticle Pt/SiO_2_ catalyst (*d*_NP_ = 10 nm; catalyst characterization shown in Figure S17; representative *Q*–*V* plots shown in Figure S19), while the plasma power ratios remained <1 at all temperatures for the 2 nm analog. Although Pt nanoparticle size altered the plasma power, the thermal apparent activation barriers for DMR were nearly identical for the 2 and 10 nm Pt/SiO_2_ catalysts (90 ± 5 and 91 ± 5 kJ/mol, respectively; Figure S18e), indicating that increasing Pt nanoparticle size did not significantly alter the apparent thermal DMR activation barrier. Nevertheless, the 10 nm Pt/SiO_2_ catalyst exhibited slightly lower CO turnover rates and slightly higher H_2_ turnover rates during thermal DMR relative to the 2 nm analog (Figure S18c,d), resulting in H_2_/CO ratios >1 (Figure S15a). This behavior is consistent with enhanced CH_4_ dehydrogenation (CH_4_→C_(s)_ + 2H_2_) on the larger Pt nanoparticles, which contain a greater fraction of Pt terrace sites (0.89 versus 0.63; Table 1). Consistent with enhanced methane dehydrogenation, thermal deactivation was also more severe for 10 nm Pt/SiO_2_, with global deactivation rate constants of 2.66 h^–1^ for CO and 3.73 h^–1^ for H_2_ compared to 0.82 and 0.88 h^–1^, respectively, for the 2 nm Pt/SiO_2_ catalyst (Figure S18a,b). The increasing H_2_/CO ratio with temperature observed for the 10 nm Pt/SiO_2_ catalyst under plasma-assisted DMR (Figure S15c, dashed black) may similarly reflect enhanced CH_4_ activation on the greater fraction of Pt terrace sites. This interpretation is consistent with the stronger C_2_H_6_ formation observed for the large Pt nanoparticles, suggesting that plasma-assisted CH_4_ activation and subsequent dehydrogenation/coupling pathways become increasingly favored relative to reforming pathways as temperature increases.

We postulate that small and abundant Pt nanoparticles for Pt/Al_2_O_3_ (2 nm) and Pt/SiO_2_ (2 nm) induce a significant interfacial polarization due to their high interfacial surface areas,^68^ which has been shown to lower the effective permittivity of the surface due to electric-field screening.^69^ In turn, this can lead to lower power dissipation, as described in the previous section. Previous studies have also shown that well-dispersed metal nanoparticles may lower the intensity of the plasma discharge while helping to increase the plasma volume.^70^ In contrast, we postulate that large Pt nanoparticles for Pt/3A (3.5 nm) and Pt/SiO_2_ (10 nm) may act as plasma hot spots or conductors, creating local electric field gradients that enhance plasma electron density and energy.^71–73^

Although the CO, H_2_, and H_2_O product turnover rates varied in magnitude across catalysts, they exhibited two distinct reactivity regimes under plasma-assisted conditions (Figure S20), with the high- and low-temperature regimes separated at ∼600 K. Apparent activation barriers extracted from the high-temperature regime that exhibited Arrhenius-like behavior were 53 ± 14, 54 ± 6, and 40 ± 8 kJ/mol for Pt/Al_2_O_3_, Pt/SiO_2_, and Pt/3A, respectively (Figure 6a–c). Compared with the thermal apparent activation barriers measured during the same alternating thermal/plasma experiments, these values correspond to reductions of ∼40 kJ/mol for Pt/Al_2_O_3_, Pt/SiO_2_, and Pt/3A, demonstrating a substantial plasma-assisted enhancement of DMR kinetics. The thermal apparent activation barriers obtained during these experiments (92 ± 6, 92 ± 3, and 83 ± 5 kJ/mol for Pt/Al_2_O_3_, Pt/SiO_2_, and Pt/3A, respectively) differed somewhat from those obtained in the dedicated thermal experiments in Figure 2 (110 ± 12, 90 ± 5, and 90 ± 3 kJ/mol, respectively), with the largest difference observed for Pt/Al_2_O_3_. Because the DMR turnover rates were determined by correcting CO formation for the RWGS contribution based on the measured H_2_O turnover rate (eq 11), the calculated thermal DMR rates are sensitive to uncertainty in the H_2_O measurement, particularly at lower temperatures where the DMR turnover rates are smaller. To probe the effect of H_2_O on extracted barriers, the thermal DMR rates were independently calculated from combined CO and H_2_ turnover rates, which eliminates reliance on the H_2_O measurement. The resulting apparent activation barriers were 104 ± 6 and 86 ± 5 kJ/mol for Pt/Al_2_O_3_ and Pt/3A, respectively, improving agreement with the corresponding values of 110 ± 12 and 90 ± 3 kJ/mol obtained from the dedicated thermal kinetic measurements (Figure 2). Differences in experimental protocol and catalyst history, including intermittent plasma exposure, may also contribute to the remaining differences. Thus, the thermal activation barriers in Figures 2 and 6 are interpreted within their respective experimental series, and differences between them are not taken as evidence for a change in the thermal DMR kinetics.

**Figure 6:**
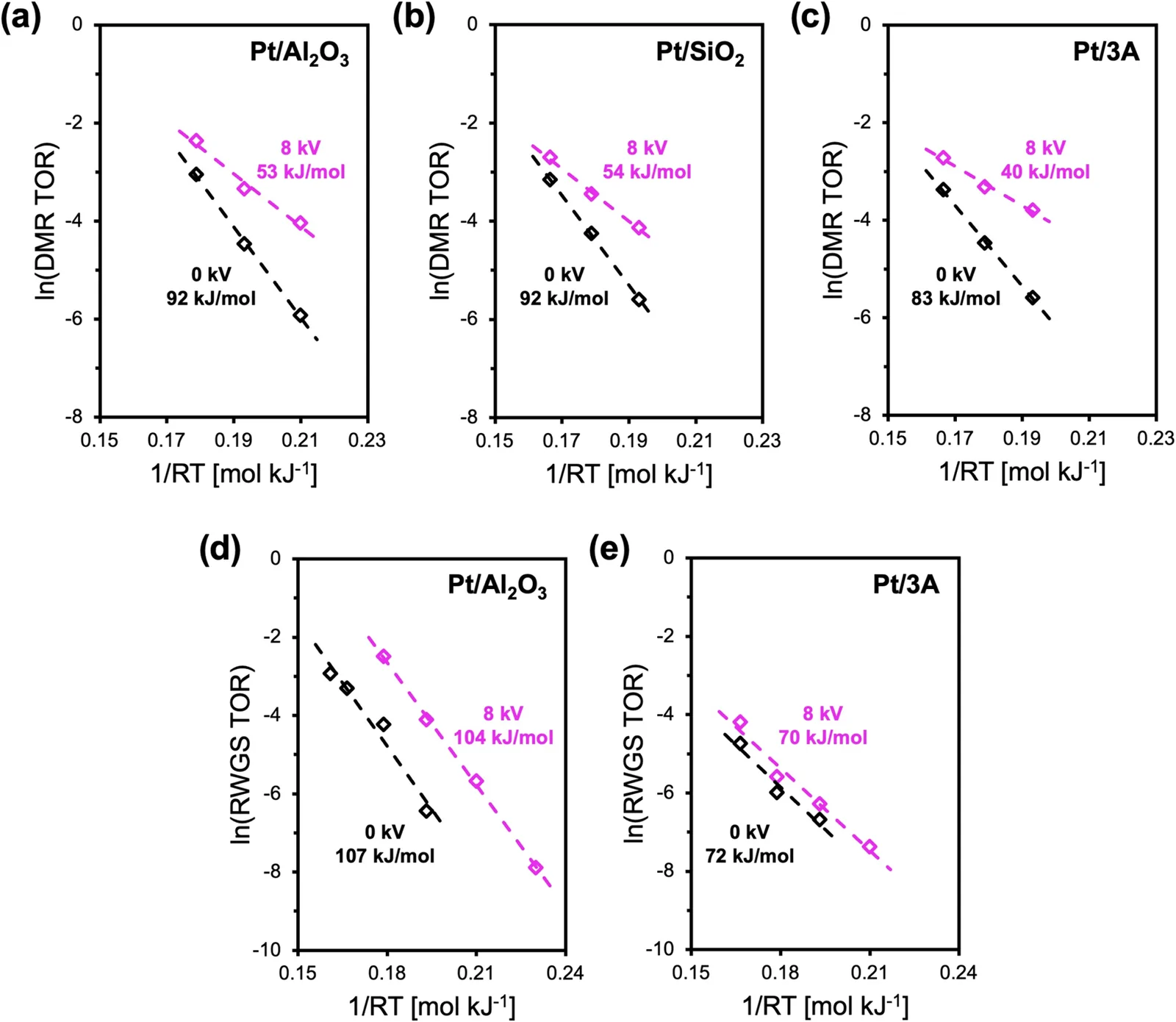
Arrhenius plots with extracted apparent activation barriers for DMR with (pink) and without (black) 8 kV plasma for (a) Pt/Al_2_O_3_, (b) Pt/SiO_2_, and (c) Pt/3A. Arrhenius plots with extracted apparent activation barriers for RWGS with (pink) and without (black) 8 kV for (d) Pt/Al_2_O_3_ and (e) Pt/3A. Reaction conditions: P_CH4_ = P_CO2_ = 6 Torr, P_Ar_ = 3 Torr, and 100 sccm total flow.

In contrast, apparent activation barriers for RWGS on Pt/Al_2_O_3_ and Pt/3A were less sensitive to plasma (Figure 6d–e), indicating the plasma did not appear to affect the kinetics of the RWGS pathway. Negligible H_2_O formation on Pt/SiO_2_ in the presence of plasma is consistent with the lack of basic sites on SiO_2_ as discussed previously and further indicates that the plasma alone did not provide measurable rates of RWGS at the conditions tested. The decrease in apparent activation barrier for DMR for all three supported Pt catalysts suggests that plasma activation influences the rate-determining step or surface coverage of reactive intermediates, but the similar apparent activation barriers for RWGS with and without the presence of plasma indicate that the RWGS pathway is less sensitive to the presence of plasma than for DMR. These results demonstrate that the NTP may selectively enhance DMR pathways on the catalyst by increasing the plasma/catalyst synergy that is affected by support properties and Pt dispersion.

During NTP-assisted DMR (V = 8 kV, P_CH4_ = P_CO2_ = 6 Torr, P_Ar_ = 3 Torr, 100 sccm total flow), C_2_H_6_ turnover rates increased with temperature for Pt/SiO_2_ and Pt/3A but decreased for Pt/Al_2_O_3_ (Figure 4c). The C_2_H_6_ formation rate ratio, defined as the rate on the supported Pt catalyst divided by the rate on the metal-free support, was slightly above 1.0 for all catalysts at temperatures below 500 K (Figure 5b), indicating (modest) enhancement of direct methane coupling with Pt. Enhanced C_2_ product formation with Pt has been observed in a related plasma-assisted catalytic study, which was attributed to Pt hydrogenation activity.^74^ However, the C_2_H_6_ formation rate ratio decreased with temperature for all catalysts and dropped below 1 above 650 K for all catalysts with relatively smaller Pt nanoparticles (< 5 nm). The most pronounced decrease in the C_2_H_6_ formation rate ratio was observed for Pt/Al_2_O_3_, followed by Pt/SiO_2_ (2 nm) and Pt/3A, while the C_2_H_6_ formation rate ratio remained above 1 over the entire temperature range for the large NP Pt/SiO_2_ (10 nm) catalyst. The apparent suppression of net C_2_H_6_ formation at elevated temperatures may arise from (i) enhanced conversion of C_2_ intermediates; (ii) increased surface coverage by oxygen-containing species that inhibit C–C coupling; or (iii) suppression of plasma-induced or surface-induced methyl radical recombination pathways. Since similar C_2_H_6_ formation rates were observed with and without Pt on the supports at low temperature, Pt likely has only a minor influence on C_2_H_6_ formation. Instead, suppressed net C_2_H_6_ formation rates at elevated temperatures on Pt catalysts are consistent with enhanced C_2_H_6_ conversion on Pt, potentially via hydrogenolysis with chemisorbed hydrogen. C_2_H_6_ hydrogenolysis on Pt has been shown to preferentially occur at Pt step and edge sites,^75^ which are more abundant on small Pt nanoparticles. Accordingly, the stronger suppression of C_2_H_6_ rates observed on Pt/Al_2_O_3_ and Pt/SiO_2_ (2 nm) relative to Pt/3A and Pt/SiO_2_ (10 nm) is consistent with their smaller Pt nanoparticles.

Consistent with this interpretation, CH_4_ selectivities for DMR were generally higher for Pt/Al_2_O_3_ relative to Pt/SiO_2_ (2 nm), Pt/3A, and Pt/SiO_2_ (10 nm) (Figure S21), reflecting greater suppression of C_2_H_6_ formation pathways. CH_4_ selectivity to DMR increased with temperature for Pt/Al_2_O_3_, Pt/SiO_2_ (2 nm), and Pt/3A, indicating that reforming increasingly dominates over methyl radical recombination at elevated temperatures. In contrast, CH_4_ selectivity to DMR decreased with temperature for Pt/SiO_2_ (10 nm), consistent with the enhanced C_2_H_6_ formation observed for the larger Pt nanoparticles at elevated temperatures. While these results demonstrate that support properties and Pt nanoparticle size strongly influence plasma-assisted DMR activity and selectivity, these parameters may also influence catalyst stability, and catalyst deactivation may therefore contribute to the measured turnover rates during these experiments. The kinetic comparisons were performed over short and consistent reaction time intervals, limiting the influence of progressive deactivation on the relative trends discussed above. Nevertheless, catalyst stability must also be considered when assessing catalyst performance.

### 3.4 Catalyst Deactivation during Plasma-Assisted DMR

Catalyst deactivation during DMR may arise from coking and/or metal nanoparticle sintering. Although Pt is comparatively resistant to coking relative to other metals active for DMR, coke formation, primarily via methane decomposition, is generally considered the main source of deactivation for thermal DMR on supported Pt catalysts.^76,77^ As discussed in Section 3.1, kinetic measurements revealed catalyst-dependent deactivation during thermal DMR, with the most pronounced deactivation observed for Pt/SiO_2_, less deactivation for Pt/3A, and no measurable deactivation for Pt/Al_2_O_3_. To investigate whether differences in carbon accumulation may contribute to these trends, carbon deposition was evaluated across the catalyst series following the shorter duration plasma-assisted DMR experiments used for kinetic analysis (Figure 7). Original TG profiles, difference TG profiles, and quantitative coke analysis are provided in Figure S23. Because the spent catalysts experienced substantially different CH_4_ conversions, coke contents were evaluated on mass, surface-area, and molar-CH_4_-conversion bases (Figure S23g). For (Pt/)­SiO_2_ (Figure 7a), a negative DTG feature near 750 K relative to the fresh material was observed primarily on spent SiO_2_ (shaded orange), consistent with the removal of easily oxidizable carbon species, such as amorphous carbon.^78^ A much smaller negative feature near 650 K was observed for spent Pt/SiO_2_ (shaded pink). Integration of these mass-loss features yielded coke contents of 3.8 and 1.6 mg-coke g-cat^–1^ for SiO_2_ and Pt/SiO_2_, respectively (Figure S23d,g). The corresponding molar ratio of coke deposited to CH_4_ converted decreased from 0.485 mol-C mol-CH_4_^–1^ for SiO_2_ to 0.014 mol-C mol-CH_4_^–1^ for Pt/SiO_2_ (Figure S23g), demonstrating substantially lower carbon accumulation relative to the extent of CH_4_ conversion upon addition of Pt. This suggests that Pt facilitates removal of carbonaceous intermediates by promoting reaction between surface-bound CH_*x*_ species and activated CO_2_-derived species, suppressing rapid dehydrogenation to form coke.^76^ The lower combustion temperature of carbon deposited on Pt/SiO_2_ relative to SiO_2_ may also reflect facilitated oxidation in the presence of Pt, which can adsorb and activate O_2_, although differences in carbon structure or proximity to Pt sites may contribute.

**Figure 7:**
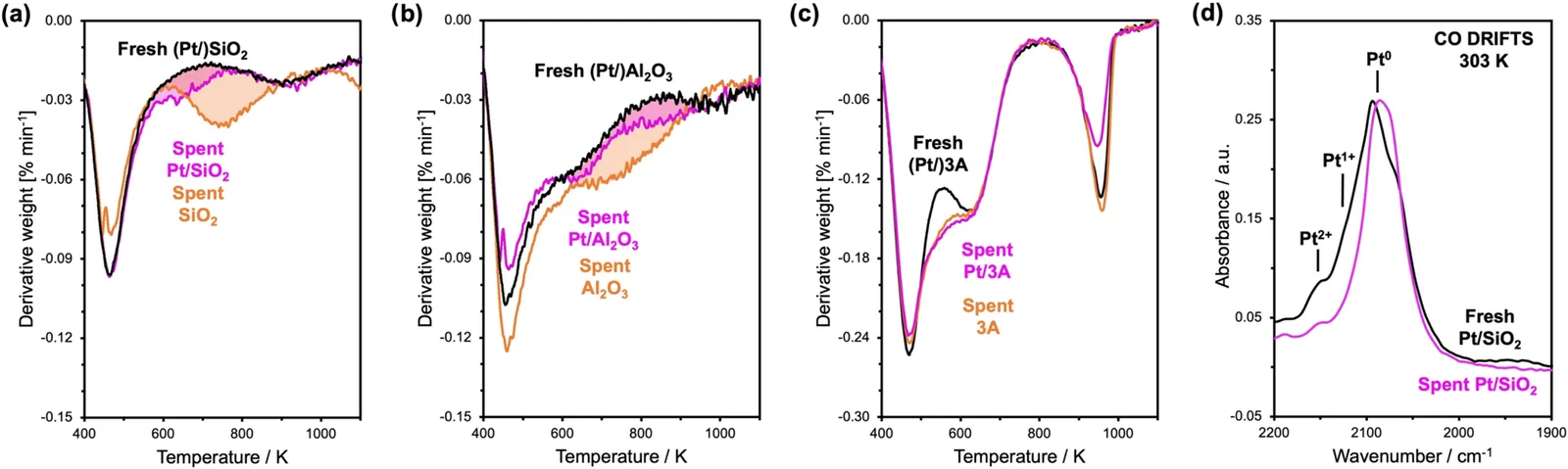
DTG profiles during TGA combustion for (a) (Pt/)­SiO_2_, (b) (Pt/)­Al_2_O_3_, and (c) (Pt/)­3A catalysts before and after a typical plasma-assisted DMR reaction, with fresh materials in black, spent metal-free supports in orange, and spent supported Pt catalysts in pink. (d) CO DRIFTS of Pt/SiO_2_ before (black) and after (pink) plasma-assisted DMR reaction. The reduced catalyst before CO flow was used as the baseline.

Similar trends were observed for (Pt/)­Al_2_O_3_ (Figure 7b), for which coke contents decreased from 4.7 mg-coke g-cat^–1^ on spent Al_2_O_3_ to 2.0 mg-coke g-cat^–1^ on spent Pt/Al_2_O_3_ (Figure S23e,g). When normalized by BET surface area, the corresponding coke contents were 17 and 7 μg-coke m^–2^ for Al_2_O_3_ and Pt/Al_2_O_3_, respectively, compared with 14 and 6 μg-coke m^–2^ for SiO_2_ and Pt/SiO_2_ (Figure S23g). Thus, the slightly greater carbon accumulation on the Al_2_O_3_-based materials persisted after accounting for differences in support surface area. The slightly higher carbon accumulation on (Pt/)­Al_2_O_3_ relative to (Pt/)­SiO_2_ may therefore be attributed to differences in surface properties, such as the higher density of basic sites on Al_2_O_3_ that stabilize carbonaceous intermediates and facilitate C–C coupling.^79^ The molar ratio of coke deposited to CH_4_ converted also decreased from 0.255 to 0.008 mol-C mol-CH_4_^–1^ with addition of Pt (Figure S23g), showing that Pt greatly decreased the fraction of converted CH_4_ retained as deposited carbon. Spent Pt/Al_2_O_3_ also exhibited a carbon oxidation feature at a higher temperature of 850 K that was not prominent for Pt/SiO_2_ (Figure S23e). This feature is consistent with ordered, graphitic carbon species that require metal sites to form on the catalyst.^78,80^ These species may form on Pt/Al_2_O_3_ due to spillover of carbonaceous species from Pt sites to basic Al_2_O_3_ sites that stabilize those species.^77,81,82^ This observation would be consistent with C_2_H_6_ turnover rates that decreased with temperature during NTP-assisted DMR on Pt/Al_2_O_3_, because the formation of graphitic carbon at elevated temperatures likely diverted CH_*x*_ intermediates away from recombination pathways that form C_2_H_6_ and instead promoted their progressive dehydrogenation and incorporation into more ordered carbon structures. This interpretation is consistent with prior studies suggesting that metal nanoparticles can scavenge reactive plasma radicals,^67^ which in the present system may decrease methyl radical recombination in the gas-phase while promoting surface-mediated dehydrogenation pathways that lead to graphitic carbon formation.

DTG profiles for fresh and spent (Pt/)­3A were convoluted with water desorption features at 470 and 625 K arising from the hygroscopic nature of the zeolite (Figures 7c and S23c,f). Above 625 K, the difference DTG profiles for 3A and Pt/3A did not exhibit clearly resolved carbon oxidation features above the baseline uncertainty, preventing reliable quantification of deposited carbon (Figure S23f,g). The absence of resolvable oxidation features indicates substantially less carbon accumulation on 3A-based materials compared to SiO_2_- and Al_2_O_3_-based materials. This behavior is consistent with the lower reaction rates and significantly smaller BET surface area of 3A (30 m^2^/g), which provides less available surface area for carbon accumulation.

Given the comparatively strong deactivation observed for Pt/SiO_2_ during the kinetic measurements, separate 6 h time-on-stream measurements were performed at 723 K to directly compare catalyst stability under thermal and plasma-assisted DMR conditions (Figure S22). The relatively inert SiO_2_ support also minimizes contributions from support-mediated reaction pathways (e.g., RWGS), enabling a more direct comparison of thermal and plasma-assisted behavior. Consistent with the deactivation observed during the kinetic measurements, CO and H_2_ turnover rates decreased with time on stream under thermal and plasma-assisted conditions (Figure S22a). Deactivation rate constants obtained from the first hour of the thermal stability measurements were 0.86 and 0.87 h^–1^ for CO and H_2_, respectively, in close agreement with the corresponding global deactivation rate constants (*k*_d_^*’*^) of 0.82 and 0.88 h^–1^ determined from the kinetic measurements (Figure S22d). This agreement further supports the global deactivation correction applied to the thermal kinetic data. Under plasma-assisted DMR, the corresponding *k*_d_^*’*^ values were 0.75 and 0.50 h^–1^ for CO and H_2_, respectively, indicating mitigation of catalyst deactivation under plasma conditions. Consistent with this trend, after 6 h on stream, plasma-assisted DMR retained 13.3 and 19.0% of the initial CO and H_2_ activities, respectively, whereas thermal DMR retained only 7.7% of the initial activity for both CO and H_2_. The applied voltage and plasma power remained approximately constant throughout the 6 h plasma-assisted experiment (Figure S22b), indicating that the observed decrease in product formation rates was not attributable to changes in the electrical characteristics of the discharge. In contrast to declining syngas formation rates, the C_2_H_6_ turnover rate initially increased before reaching a relatively stable value after approximately 2 h of plasma operation (Figure S22c). This behavior may reflect a shift toward plasma-phase CH_3_ radical recombination as Pt sites responsible for surface-mediated reforming progressively deactivate, while concurrent carbon accumulation on the catalyst surface may further alter C_2_H_6_ formation pathways.

Carbon deposition following the 6 h stability experiments was quantified by oxidative TGA (Figure S24). The normalized mass loss profiles and corresponding DTG profiles for fresh Pt/SiO_2_ and Pt/SiO_2_ following thermal and plasma-assisted DMR are shown in Figure S24a–b. Coke contents of 8.4 ± 0.6 and 10.1 ± 0.4 mg-C g-cat^–1^ were measured following thermal and plasma-assisted DMR, respectively. Although the absolute coke contents after 6 h on stream were similar under thermal and plasma-assisted conditions, substantially more CH_4_ was converted during the plasma-assisted reaction. Integration of CH_4_ conversion over the full time-on-stream yielded cumulative amounts of CH_4_ converted of 8.1 ± 0.2 and 29.1 ± 0.3 mmol under thermal and plasma-assisted conditions, respectively. The corresponding molar ratios of coke deposited to CH_4_ converted were 0.072 ± 0.005 and 0.024 ± 0.001 mol-C mol-CH_4_^–1^, respectively (67% difference), demonstrating improved coke resistance relative to the extent of reaction under plasma. The dominant carbon oxidation feature shifted from approximately 666 K following thermal DMR to 707 K following plasma-assisted DMR (Figure S24c–d), consistent with the formation of more ordered or graphitic carbon under plasma-assisted conditions. However, differences in the location of carbon deposits and their proximity to Pt sites may also contribute to the higher oxidation temperature, and further characterization would be required to distinguish between these possibilities. Post-reaction TEM showed similar Pt nanoparticle sizes following the 6 h thermal and plasma-assisted DMR experiments (1.8 ± 0.4 and 1.7 ± 0.3 nm, respectively; Figure S25), with no appreciable Pt nanoparticle growth after either condition relative to fresh Pt/SiO_2_ (1.9 ± 1.1 nm). The particle size distributions following both 6 h experiments were narrower than that of the fresh catalyst, possibly indicating agglomeration of the smallest Pt clusters and partial redispersion of larger nanoparticles under reaction conditions, consistent with the nanoparticle restructuring discussed below. Regardless, the greater retention of rates and lower coke formation normalized by the amount of CH_4_ converted during plasma-assisted DMR indicate that plasma assistance mitigates catalyst deactivation and improves coke resistance relative to thermal DMR.

Subtle Pt nanoparticle restructuring during NTP-assisted DMR was further evaluated by post-reaction CO-pulse chemisorption and CO DRIFTS (Figure S2 and Table S2). For Pt/SiO_2_, Pt dispersion measured by CO-pulse chemisorption was 0.40 and 0.34 before and after reaction, respectively, indicating minor Pt sintering. Pt nanoparticle sizes measured by TEM were 1.9 ± 1.1 and 1.6 ± 0.6 nm before and after reaction, respectively, which is also consistent with minimal sintering. We again observed a slightly narrower distribution, suggesting small Pt clusters or isolated Pt atoms on SiO_2_ appeared to agglomerate during reaction, as evidenced by the lower relative abundance of small (<1.5 nm) particles (Figure S2d). This is supported by CO DRIFTS measurements (Figure 7d), where a significant reduction in the absorbance for bands at 2150 and 2125 cm^–1^, assigned to CO adsorbed on Pt^2+^ and Pt^1+^ partially oxidized Pt species, respectively, suggests agglomeration of the smallest Pt clusters into larger Pt nanoparticles during NTP-assisted DMR. Interestingly, the relative abundance of larger (>3 nm) Pt nanoparticles also decreased after reaction, which could indicate partial redispersion of the largest nanoparticles, perhaps driven by reducing CO and H_2_ species present under reaction conditions. For Pt/3A, Pt dispersion measured by CO-pulse chemisorption was 0.27 and 0.14 before and after NTP-assisted DMR, respectively, demonstrating more severe sintering than for Pt/SiO_2_. In contrast, Pt dispersion for Pt/Al_2_O_3_ was 0.59 and 0.61 before and after NTP-assisted DMR, respectively, indicating no measurable plasma-induced sintering for this catalyst. This suggests that the large Pt nanoparticles on 3A were more susceptible to sintering than the smaller particles on Al_2_O_3_ and SiO_2_, likely reflecting differences in metal-support interactions or the lower available surface area on 3A for high Pt dispersion. Overall, these results demonstrate that the extent of Pt restructuring during NTP-assisted DMR depends strongly on the catalyst support, with substantial loss of Pt dispersion observed for Pt/3A, minor changes for Pt/SiO_2_, and no measurable loss of dispersion for Pt/Al_2_O_3_.

## 4 Conclusion

Nonthermal plasma (NTP)-assisted dry methane reforming (DMR) was investigated on Pt nanoparticles supported on Al_2_O_3_, SiO_2_, and zeolite 3A in a packed-bed dielectric barrier discharge (DBD) reactor to elucidate the interplay between plasma activation, metal nanoparticles, and catalyst supports. Thermal catalysis studies confirmed that DMR is primarily controlled by structure-sensitive CH_4_ activation that is enhanced on Pt step and edge sites. Competing reverse water–gas shift (RWGS) activity depended strongly on support identity, with significant RWGS observed on Pt/Al_2_O_3_ and Pt/3A due to enhanced CO_2_ adsorption on basic sites, while negligible RWGS activity was observed on Pt/SiO_2_.

Under plasma-assisted catalysis conditions, syngas formation rates were significantly enhanced, and apparent activation barriers for DMR were reduced by 38–43 kJ/mol across all catalysts, consistent with plasma-enhanced CH_4_ and CO_2_ activation. In contrast, RWGS apparent activation barriers were unchanged in the presence of plasma, suggesting that plasma activation preferentially enhanced reforming pathways over H_2_O formation pathways under the conditions tested. *Q*–*V* analysis demonstrated that both support dielectric properties and Pt nanoparticle size influenced plasma power and plasma/catalyst synergy despite minimal changes in the reduced electric field (*E*/*N*) of the bulk plasma. Plasma power was suppressed by small Pt nanoparticles and enhanced by larger Pt nanoparticles, suggesting competing effects of interfacial polarization and local electric field enhancement. Catalyst stability was also strongly dependent on support identity and plasma exposure. Extracted deactivation rate constants showed measurable deactivation during thermal DMR on Pt/SiO_2_, which was mitigated under plasma-assisted conditions. Post-reaction characterization further revealed that Pt suppressed coke accumulation relative to metal-free supports and that Pt restructuring was support-dependent, with Pt/Al_2_O_3_ resistant to sintering, modest restructuring of Pt/SiO_2_, and significant Pt agglomeration on 3A after plasma exposure.

Overall, these results demonstrate that activity, selectivity, and stability in plasma-assisted DMR on supported Pt catalysts are strongly influenced by a complex interplay of the plasma, plasma-surface interactions, and catalyst structure. Catalyst support properties influenced plasma properties and reaction pathways, and Pt nanoparticle size affected plasma power, CH_4_ activation, and reforming activity. These findings provide mechanistic insights for the rational design of catalysts and reactors for electrified CH_4_ and CO_2_ conversion and related plasma-assisted catalytic C_1_ upgrading processes.

## Supplementary Material



## Notes

Additional experimental data and analyses, including reactor photographs, catalyst characterization, reaction kinetics and selectivity, plasma electrical characterization, catalyst stability, and coke quantification (PDF)
