# Supplementary material for: Probing the Interface of Plasma, Metal, and Catalyst Support in Plasma-Assisted Dry Methane Reforming on Pt-Based Catalysts
Source: Energy Fuels. 2026 Sep 12;40(38):21449–63. doi: 10.1021/acs.energyfuels.6c03688 (PMC13617971; doi:10.1021/acs.energyfuels.6c03688)
Supplement: Supplementary file 1 [file ef6c03688_si_001.pdf]

Supporting Information (SI)

Probing the interface of plasma, metal, and catalyst support in plasma-assisted dry methane reforming on Pt-based catalysts

James L. Trettin[1], Janice M. Kankolongo[1], Cole W. Hullfish[1], Bruce E. Koel[1,2], and Michele L. Sarazen[1,2,]*
[1]Department of Chemical and Biological Engineering, Princeton University, Princeton, New Jersey, 08540
[2]Princeton Plasma Physics Laboratory, Princeton, New Jersey, 08540
Corresponding author: msarazen@princeton.edu

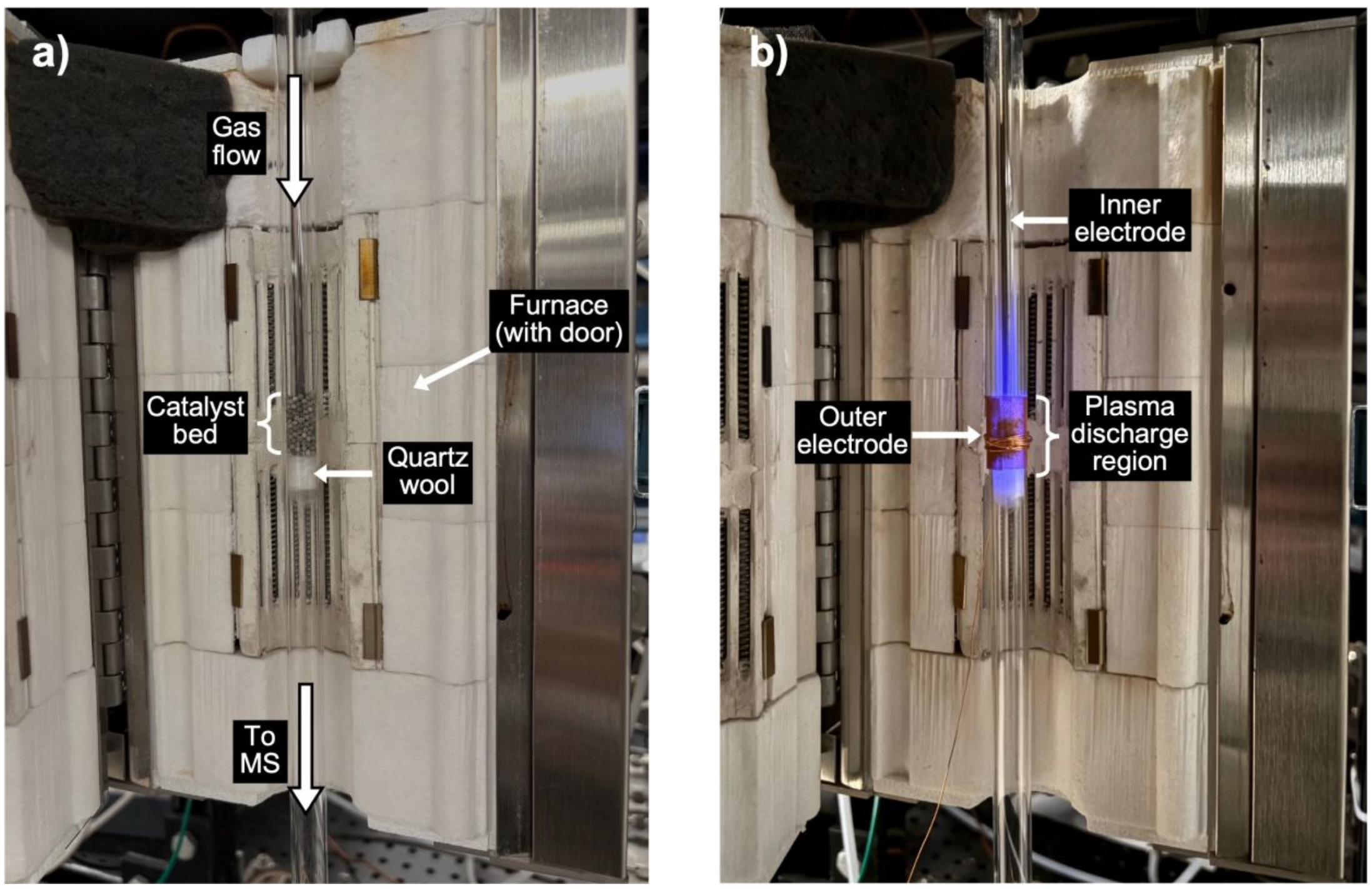

Figure S1: Photograph of the packed-bed DBD reactor used for thermal and plasma-assisted DMR. (a) Catalyst bed location and direction of gas flow. (b) Electrode configuration and plasma discharge region. The furnace door is closed during normal reactor operation.

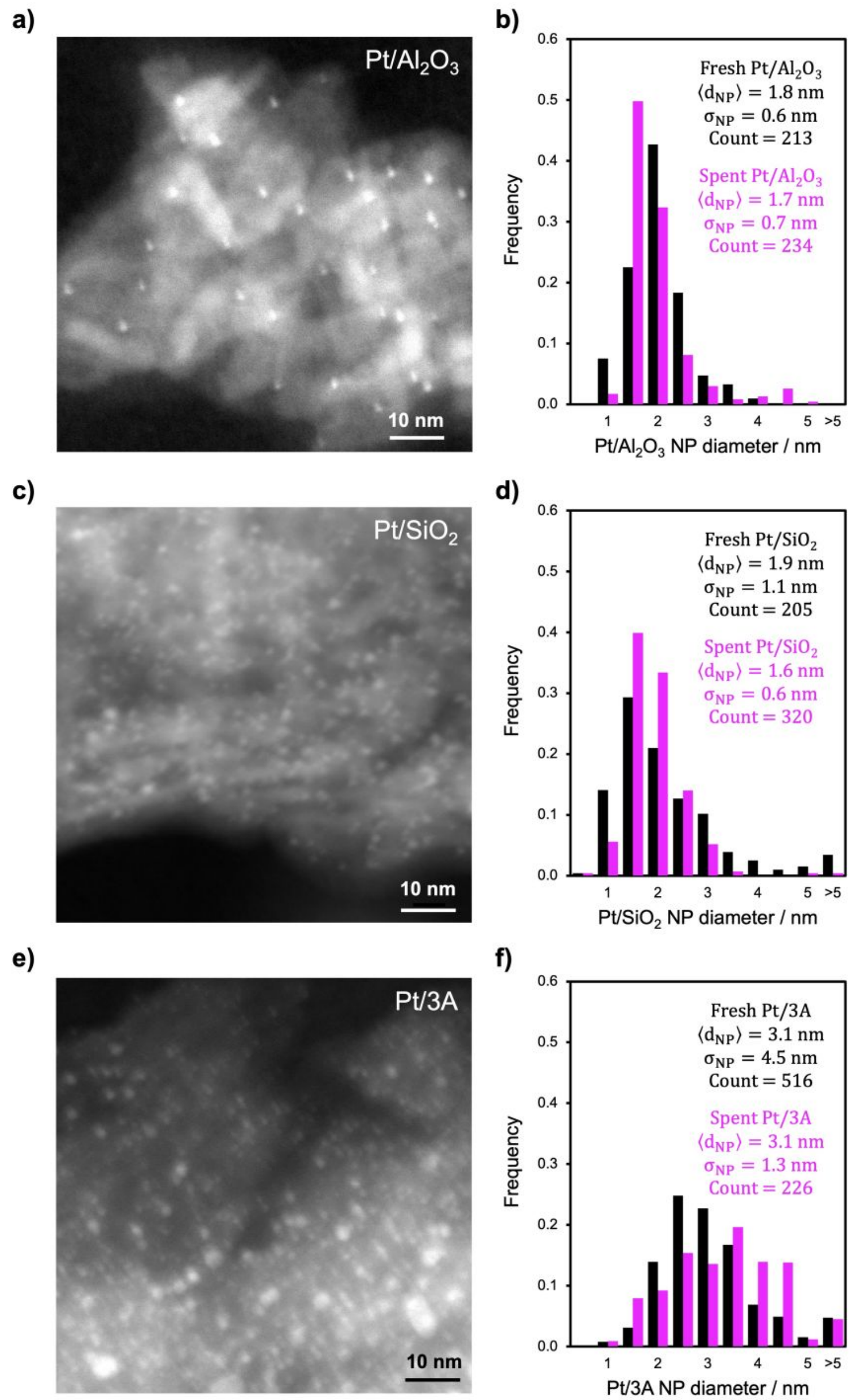

Figure S2: Representative TEM micrographs and corresponding Pt nanoparticle size histograms for fresh (black) and spent (pink) (a,b) Pt/Al_2O_3, (c,d) Pt/SiO_2, and (e,f) Pt/3A.

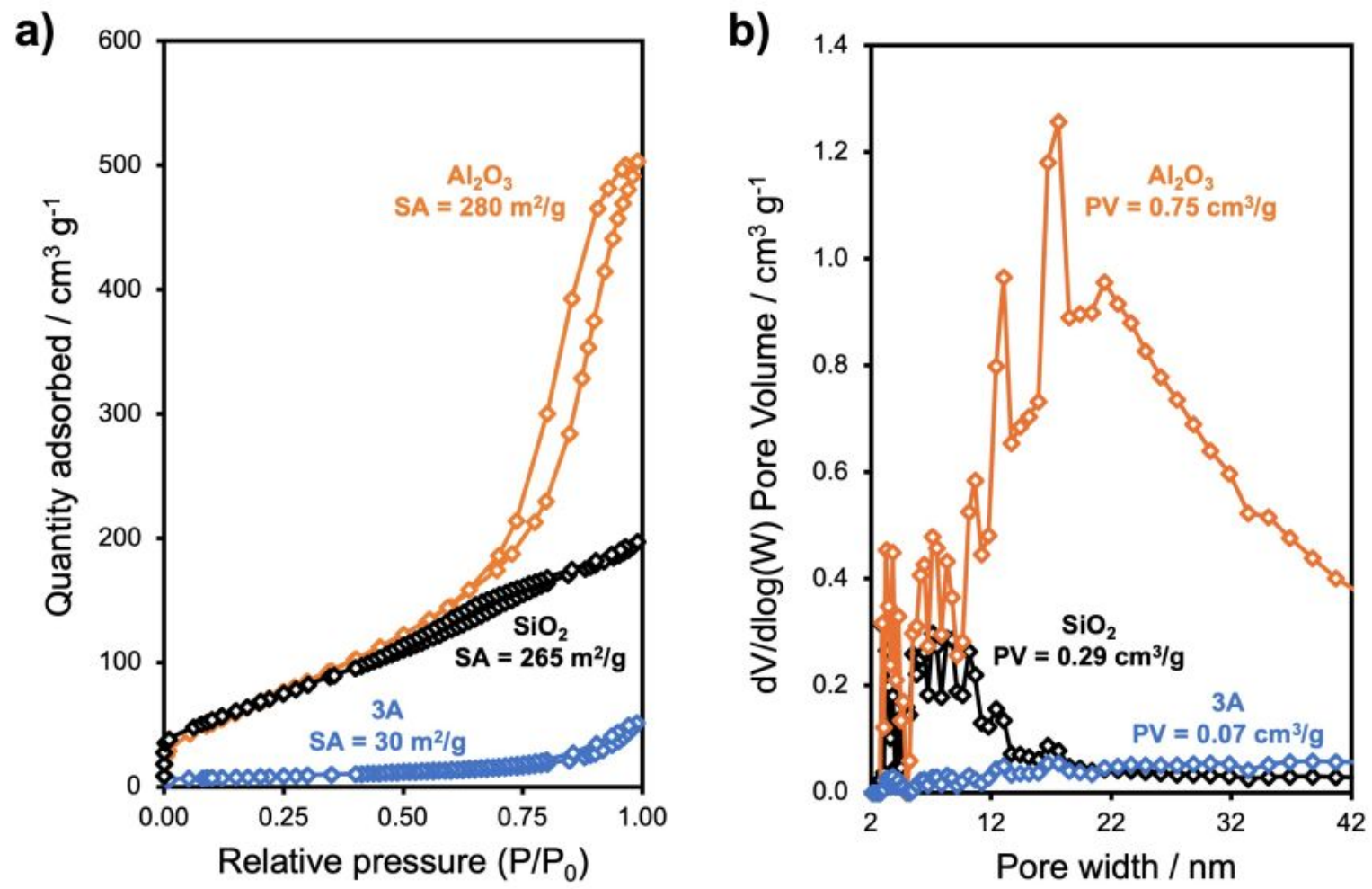

Figure S3: (a) BET isotherms and (b) BJH pore size distributions for Al_2O_3 (orange), SiO_2 (black), and zeolite 3A (blue) supports determined from N_2 physisorption measurements.

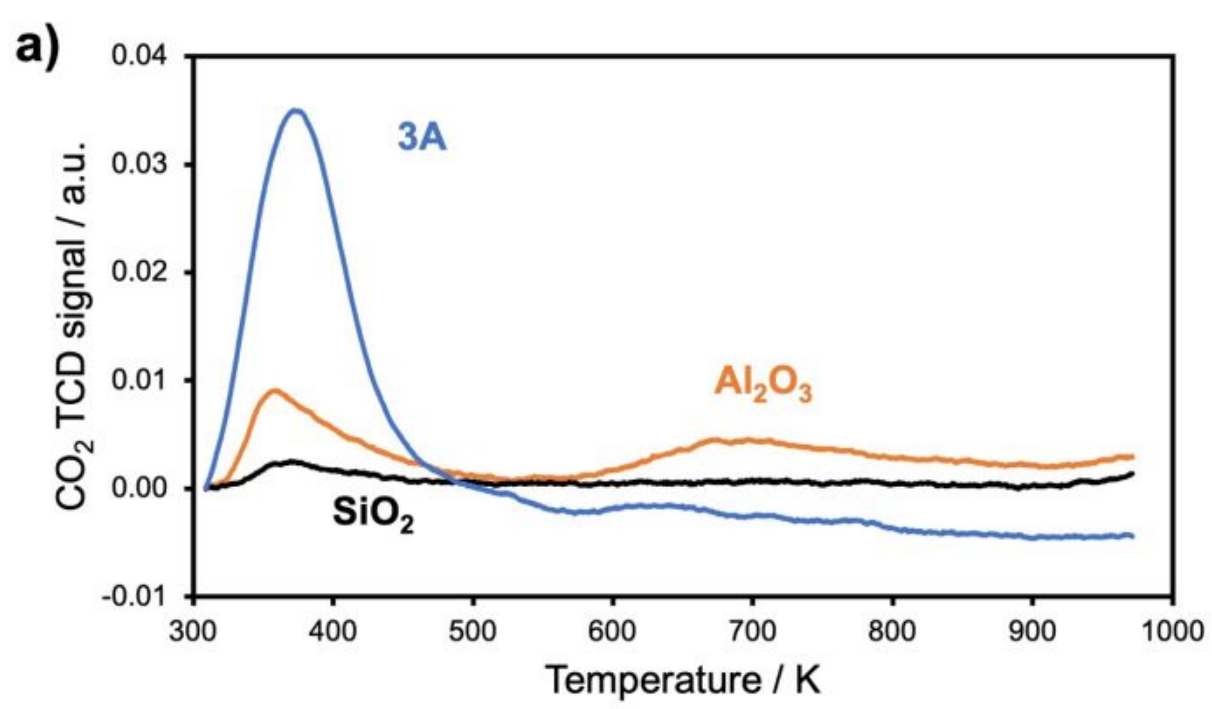

b)

Material	Peak	T at max [K]	Quantity [mmol g^{-1}]	Total quantity [mmol g^{-1}]
Al_2O_3	1	360	0.10	0.20
	2	698	0.11	
SiO_2	1	368	0.02	0.04
	2	723	0.02	
Zeolite 3A	1	372	0.33	0.36
	2	601	0.04	

Figure S4: (a) CO_2 TPD profiles for Al_2O_3 (orange), SiO_2 (black), and zeolite 3A (blue), and (b) tabulated associated low and high temperature desorption features and total mass-normalized CO_2 adsorption quantities.

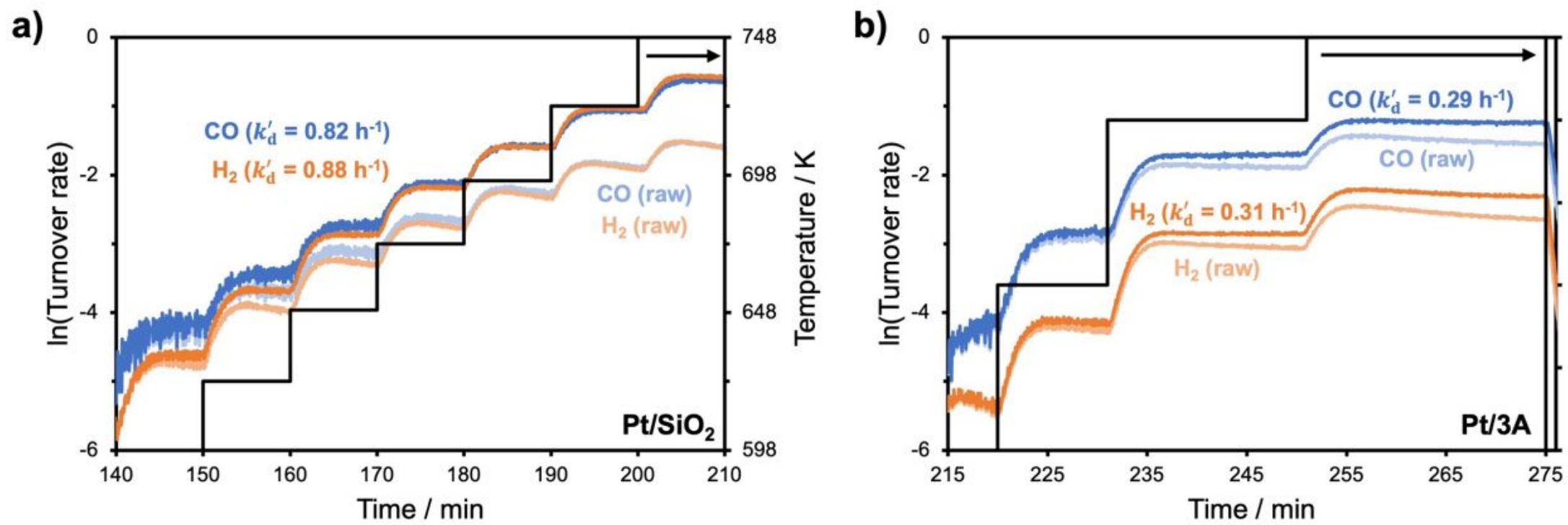

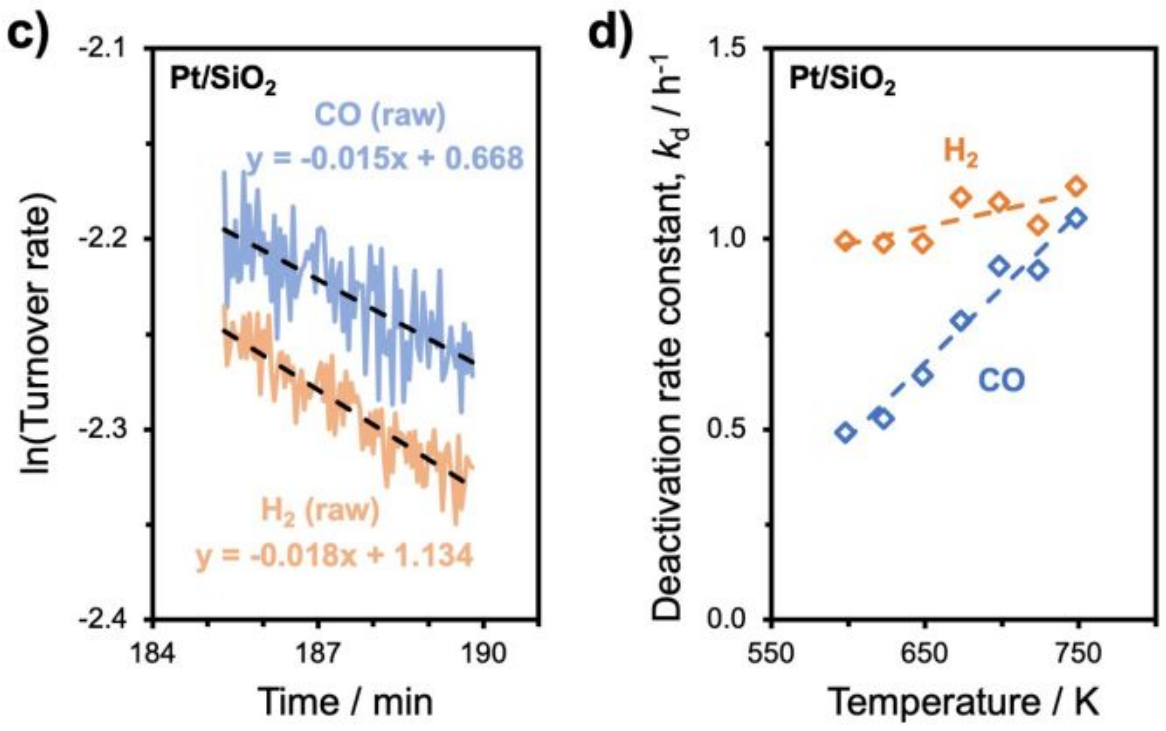

e)

Catalyst	$k'_{d,CO}$ / h⁻¹	$k'_{d,H2}$ / h⁻¹	$k'_{d,H2O}$ / h⁻¹
Pt/Al_2O_3	0.00	0.00	0.00
Pt/SiO_2	0.82	0.88	–
Pt/3A	0.29	0.31	0.34

Figure S5: As-measured turnover rates for CO (light blue) and H_2 (light orange) and deactivation-adjusted turnover rates for CO (blue) and H_2 (orange) as a function of time on stream while varying temperature (black, right axis) during thermal DMR on (a) Pt/SiO_2 and (b) Pt/3A. For Pt/SiO_2, (c) representative first-order deactivation fit over a 5 min isothermal period at 698 K used to assess the apparent deactivation rate constant, k_d, and (d) temperature dependence of the apparent deactivation rate constants extracted from these isothermal periods. (e) Global deactivation rate constants (k'_d) used to adjust thermal DMR and RWGS turnover rates.

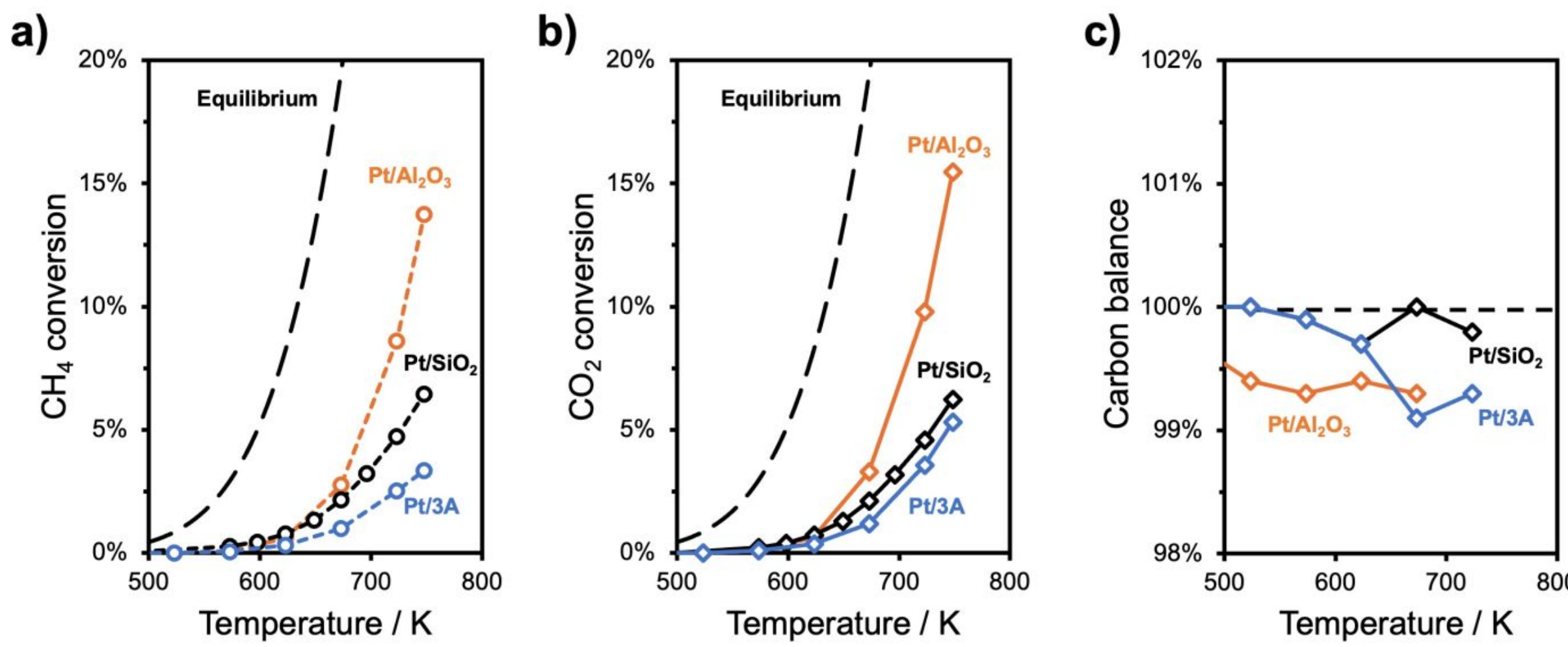

Figure S6: (a) CH_4 conversion, (b) CO_2 conversion, and (c) corresponding carbon balances during thermal DMR on Pt/Al_2O_3 (orange), Pt/SiO_2 (black), and Pt/3A (blue) as a function of temperature. The dashed line in (a) and (b) indicates the calculated DMR equilibrium conversion under the experimental reaction conditions, and the horizontal dashed line in (c) indicates complete carbon balance. Reaction conditions: $P_{CH4} = P_{CO2}$ = 6 Torr, P_{Ar} = 3 Torr, and 100 sccm total flow.

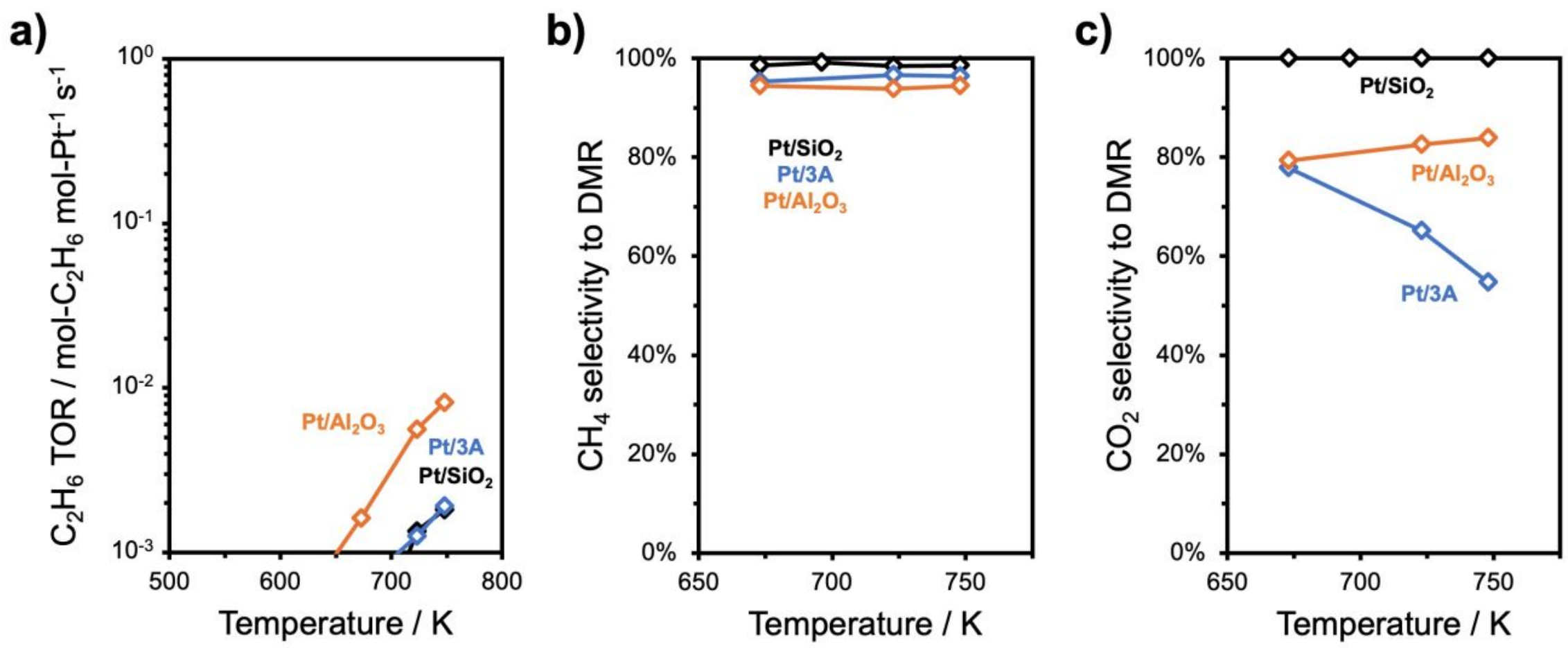

Figure S7: (a) C_2H_6 turnover rate, (b) CH_4 selectivity to DMR, and (c) CO_2 selectivity to DMR during thermal DMR on Pt/Al_2O_3 (orange), Pt/SiO_2 (black), and Pt/3A (blue) as a function of temperature.

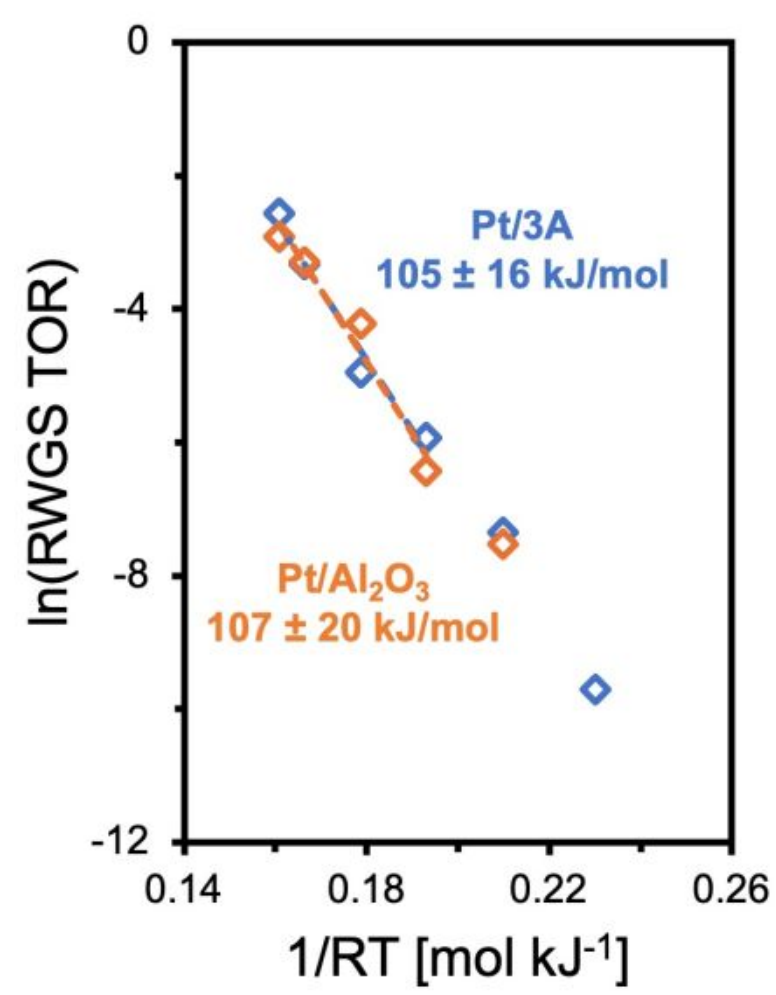

Figure S8: Arrhenius plot for RWGS during thermal DMR on Pt/Al_2O_3 (orange) and Pt/3A (blue).

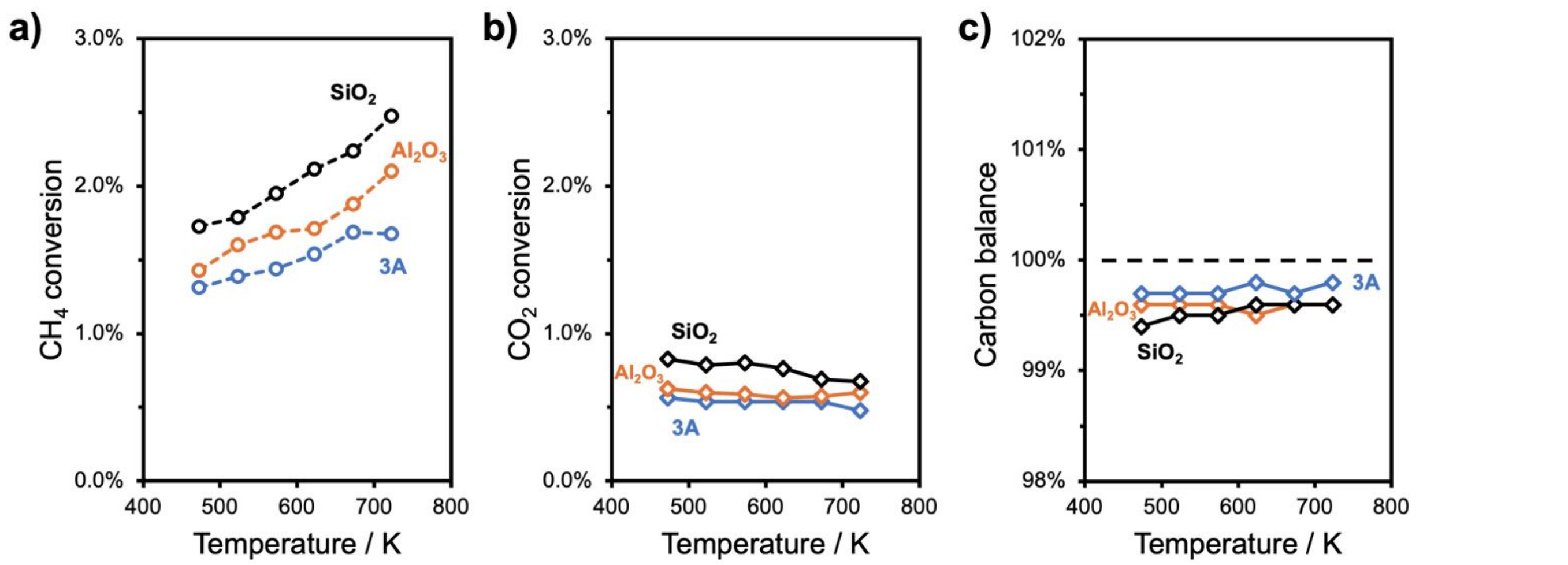

Figure S9: Conversions of (a) CH_4 and (b) CO_2, and (c) the corresponding carbon balance during plasma-assisted DMR on metal-free Al_2O_3 (orange), SiO_2 (black), and 3A (blue) as a function of temperature. The horizontal dashed line in (c) indicates complete carbon balance. All measurements were recorded at 8 kV applied voltage. Reaction conditions: $P_{CH4} = P_{CO2} = 6$ Torr, $P_{Ar} = 3$ Torr, and 100 sccm total flow.

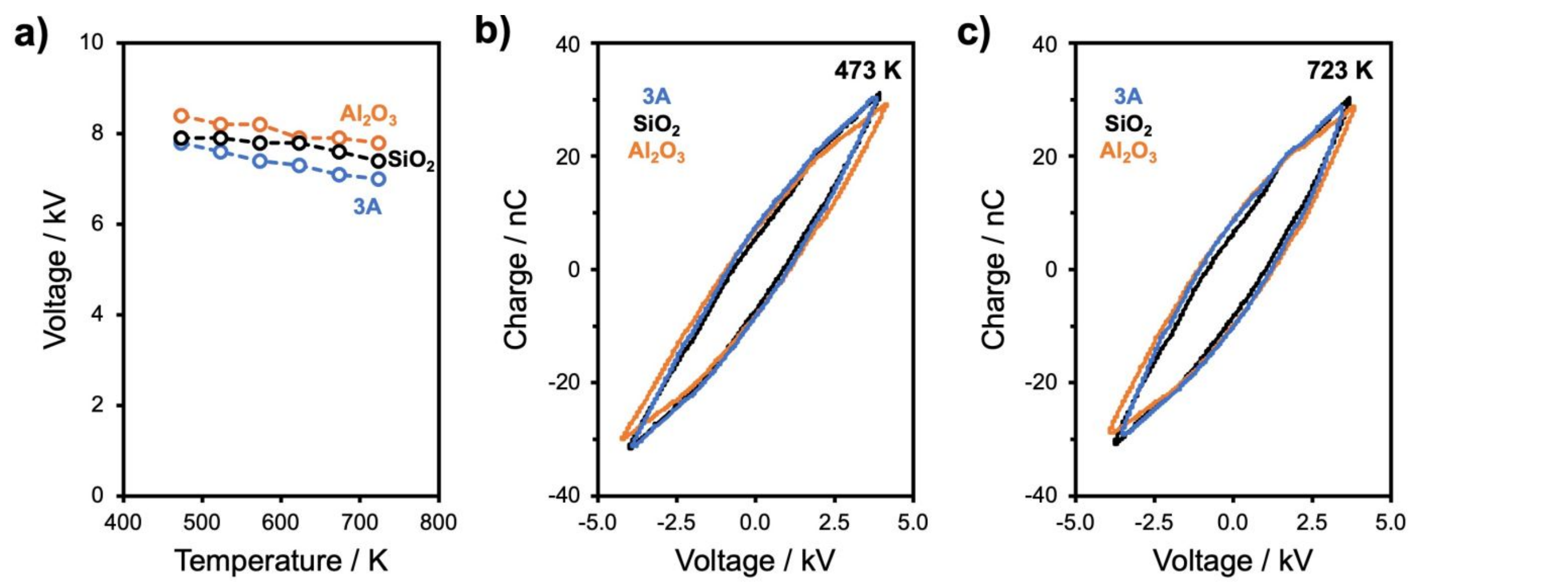

Figure S10: (a) Applied voltage as a function of temperature, and representative Lissajous (Q–V) curves at (b) 473 K and (c) 723 K for metal-free Al_2O_3 (orange), SiO_2 (black), and zeolite 3A (blue). All measurements were recorded at 8 kV applied voltage. Reaction conditions: $P_{CH4} = P_{CO2} = 6$ Torr, $P_{Ar} = 3$ Torr, and 100 sccm total flow.

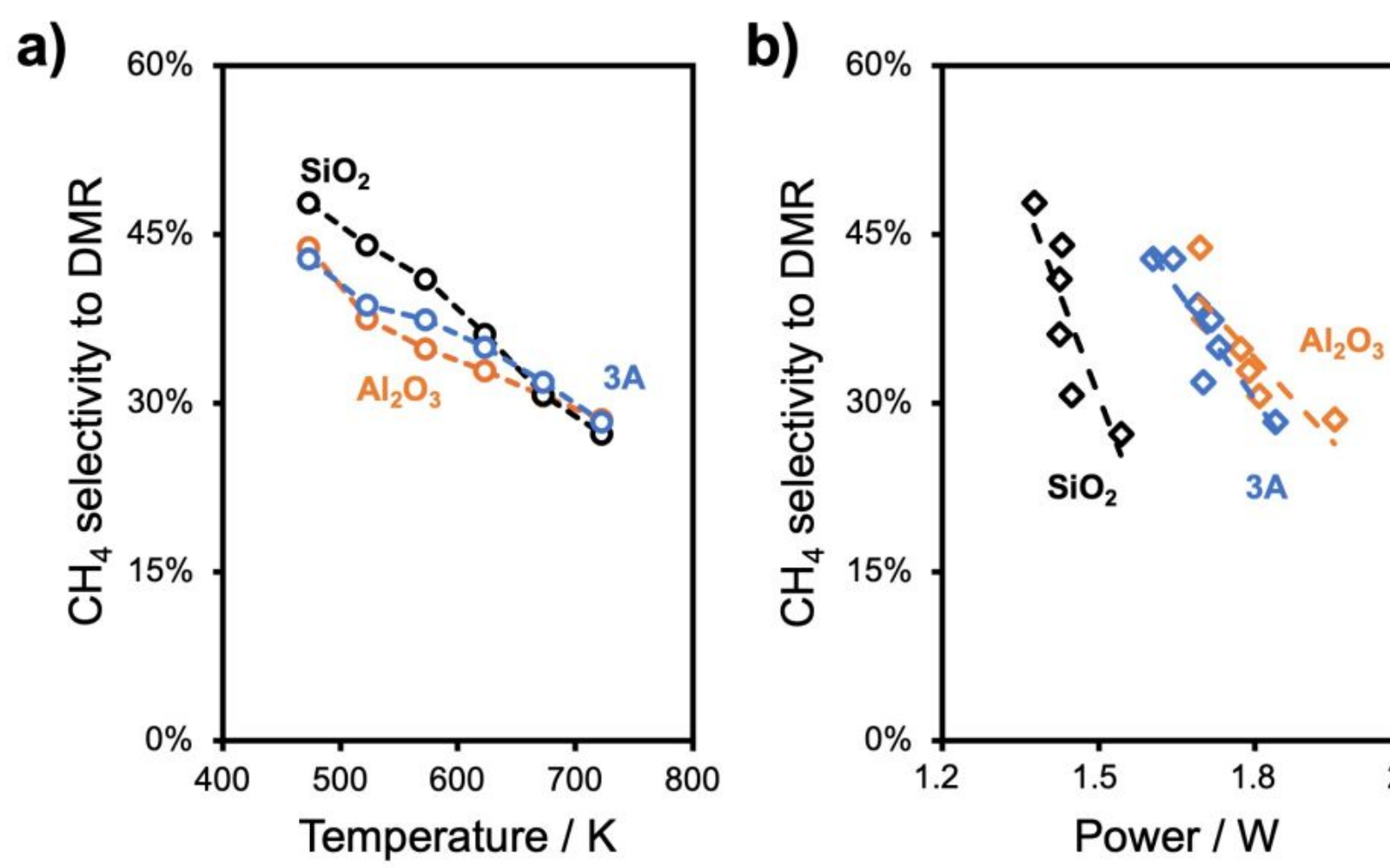

Figure S11: CH_4 selectivity to DMR as a function of (a) temperature and (b) power during plasma-assisted DMR on metal-free Al_2O_3 (orange), SiO_2 (black), and zeolite 3A (blue). All measurements were recorded at 8 kV applied voltage. Reaction conditions: $P_{CH4} = P_{CO2} = 6$ Torr, $P_{Ar} = 3$ Torr, and 100 sccm total flow.

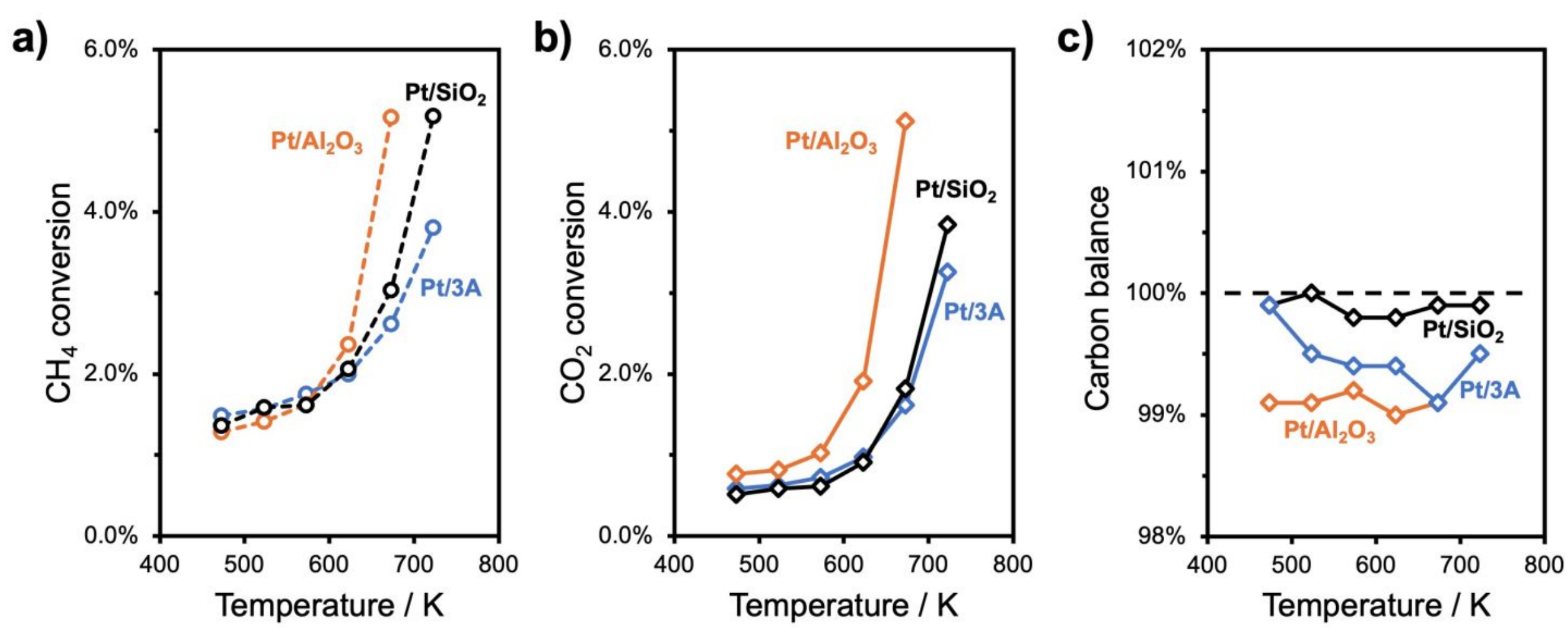

Figure S12: (a) CH_4 conversion, (b) CO_2 conversion, and (c) corresponding carbon balances during plasma-assisted DMR on Pt/Al_2O_3 (orange), Pt/SiO_2 (black), and Pt/3A (blue) catalysts as a function of temperature. The horizontal dashed line in (c) indicates complete carbon balance. All measurements were recorded at 8 kV applied voltage. Reaction conditions: $P_{CH4} = P_{CO2} = 6$ Torr, $P_{Ar} = 3$ Torr, and 100 sccm total flow.

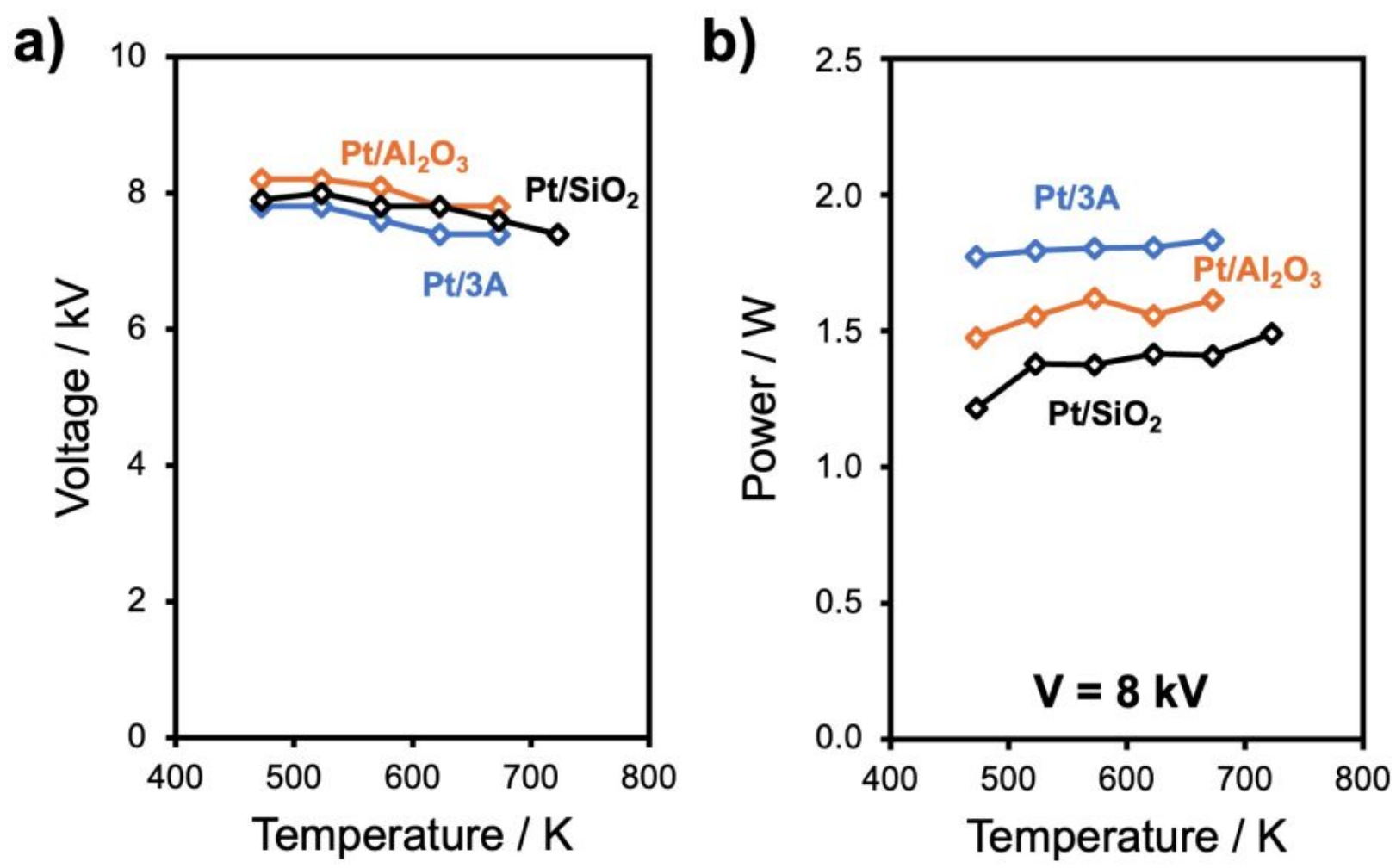

Figure S13: (a) Applied voltage and (b) plasma power during plasma-assisted DMR on Pt/Al_2O_3 (orange), Pt/SiO_2 (black), and Pt/3A (blue) as a function of temperature. Reaction conditions: $P_{CH4} = P_{CO2} = 6$ Torr, $P_{Ar} = 3$ Torr, and 100 sccm total flow.

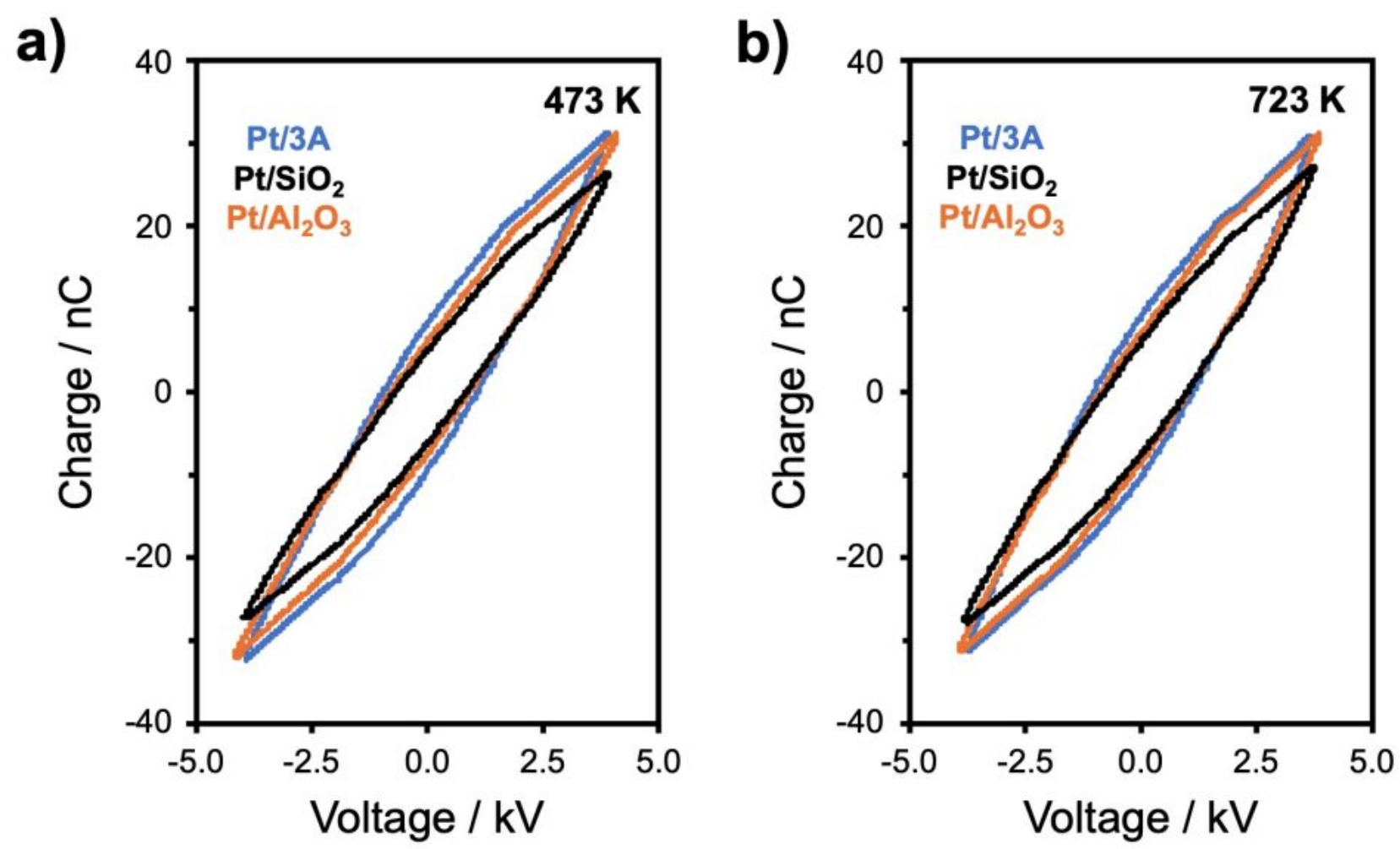

Figure S14: Lissajous (Q–V) curves at (a) 473 K and (b) 723 K during plasma-assisted DMR on Pt/Al_2O_3 (orange), Pt/SiO_2 (black), and Pt/3A (blue). Reaction conditions: $P_{CH4} = P_{CO2} = 6$ Torr, $P_{Ar} = 3$ Torr, and 100 sccm total flow at an applied voltage of 8 kV.

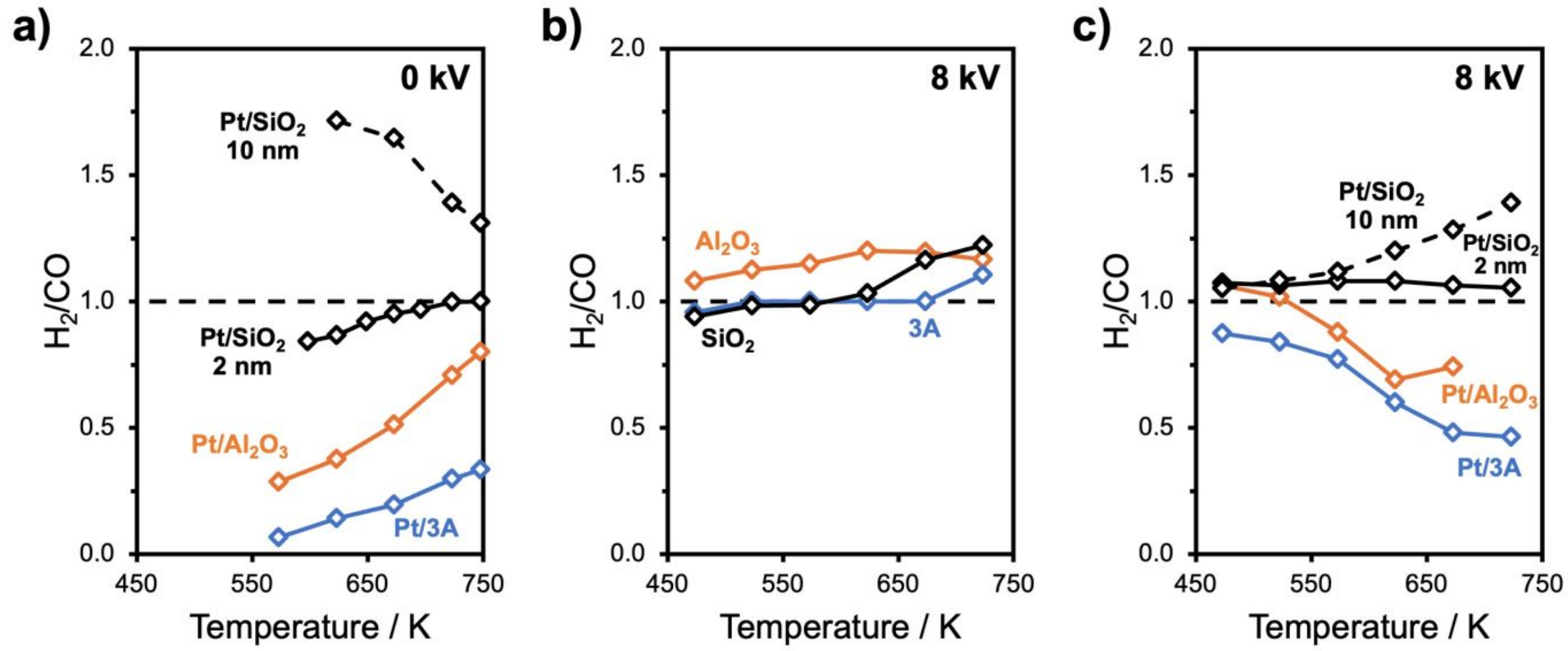

Figure S15: H_2/CO product ratios during (a) thermal DMR on supported Pt catalysts, (b) plasma-assisted DMR on metal-free supports, and (c) plasma-assisted DMR on supported Pt catalysts as a function of temperature. The horizontal dashed line at $H_2/CO = 1$ represents the stoichiometric product ratio for DMR. Plasma-assisted DMR was performed at 8 kV applied voltage. Reaction conditions: $P_{CH4} = P_{CO2} = 6$ Torr, $P_{Ar} = 3$ Torr, and 100 sccm total flow.

Table S1: Reaction performance during plasma-assisted DMR over metal-free supports and supported Pt catalysts at 673 K and an applied voltage of 8 kV. Reaction conditions: $P_{CH4} = P_{CO2} = 6$ Torr, $P_{Ar} = 3$ Torr, and 100 sccm total flow. *n.d.*, not detected.

Catalyst	X_{CH4} [%]	X_{CO2} [%]	H_2/CO	H_2O formation rate [μmol-H_2O s^{-1} W^{-1}]	C_2H_6 formation rate [μmol-C_2H_6 s^{-1} W^{-1}]	C balance [%]
Al_2O_3	1.9	0.6	1.20	n.d.	0.11	99.6
SiO_2	2.2	0.7	1.16	n.d.	0.16	99.6
3A	1.7	0.5	1.00	n.d.	0.10	99.7
Pt/Al_2O_3	5.2	5.1	0.74	0.14	0.07	99.1
Pt/SiO_2	3.0	1.8	1.06	n.d.	0.13	99.9
Pt/3A	2.6	1.6	0.48	0.02	0.09	99.1

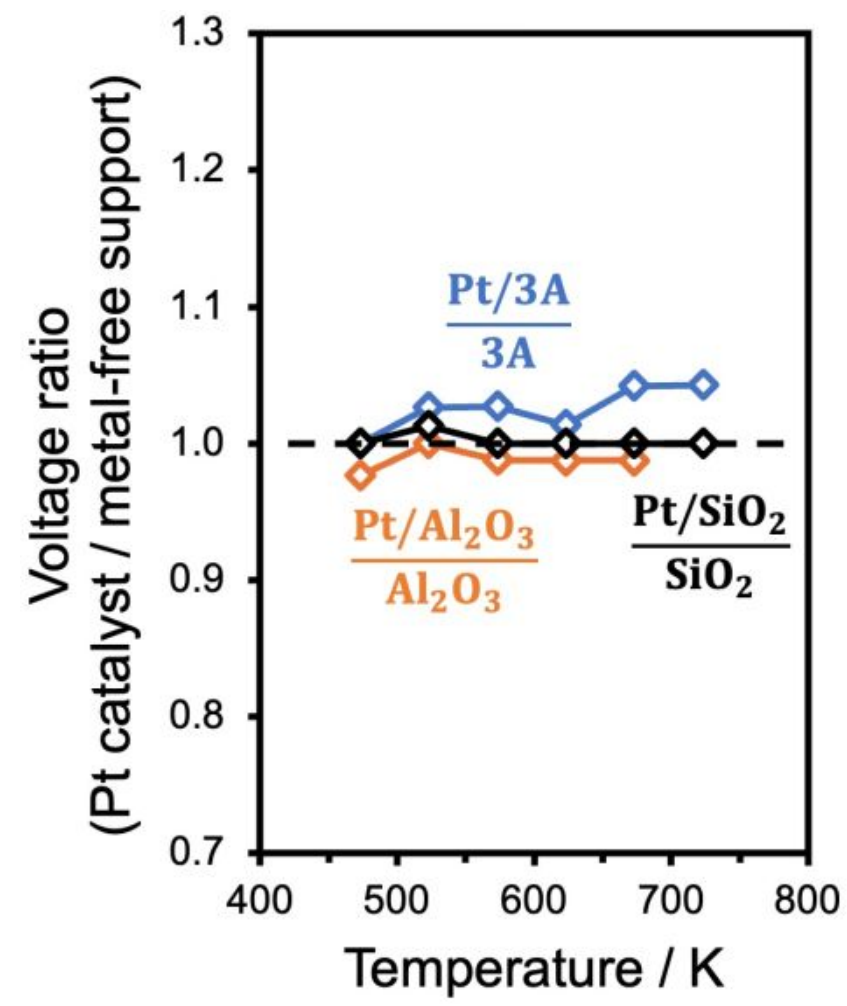

Figure S16: Voltage ratio, defined for the Pt catalyst relative to the metal-free support, during NTP-assisted DMR on (Pt/)Al_2O_3 (orange), (Pt/)SiO_2 (black) and (Pt/)3A (blue). Reaction conditions: $P_{CH4} = P_{CO2} = 6$ Torr, $P_{Ar} = 3$ Torr, and 100 sccm total flow.

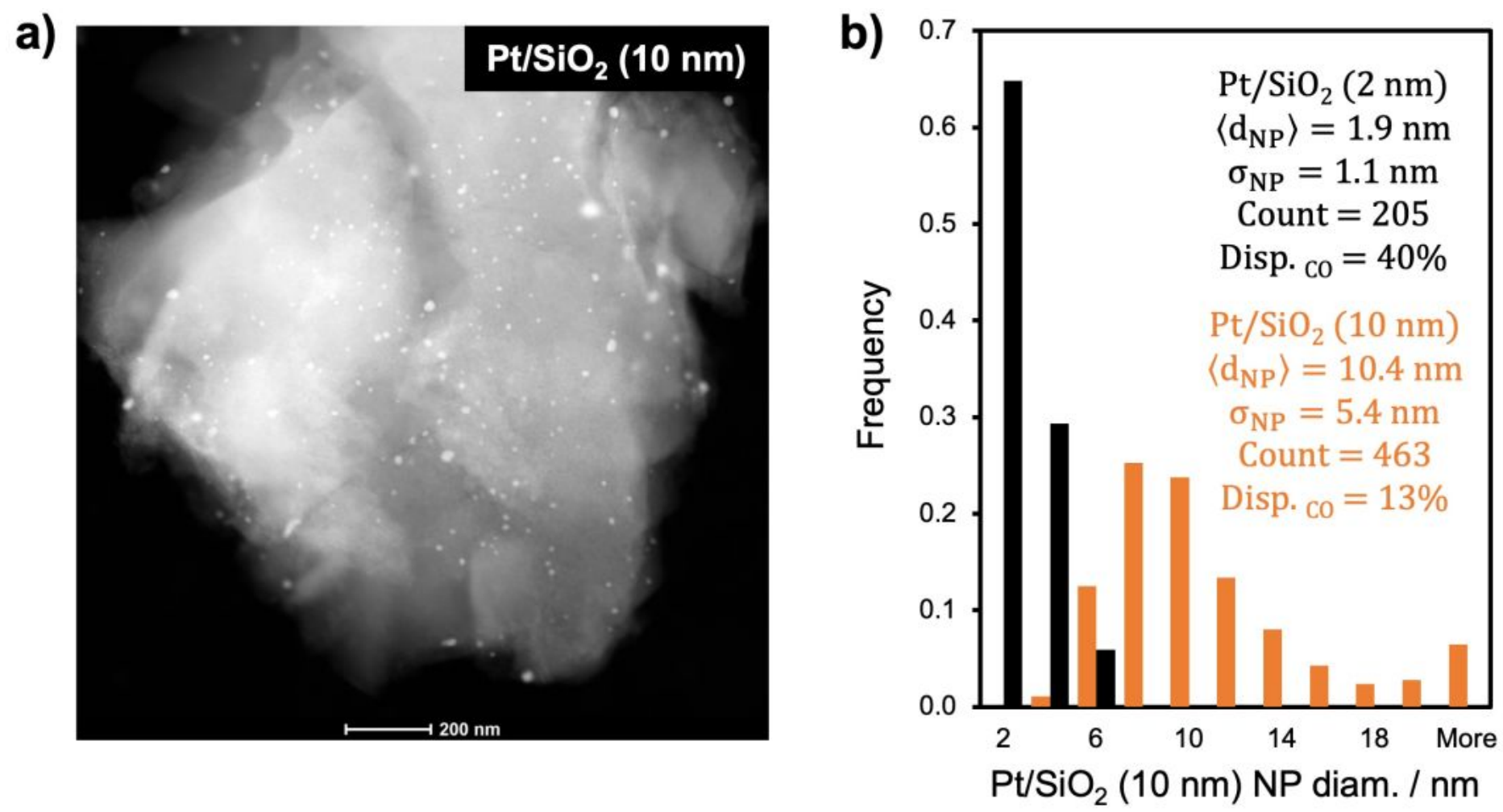

Figure S17: (a) TEM micrograph and (b) corresponding Pt nanoparticle size histograms for Pt/SiO_2 (10 nm) (orange), compared to that for Pt/SiO_2 (2 nm) (black).

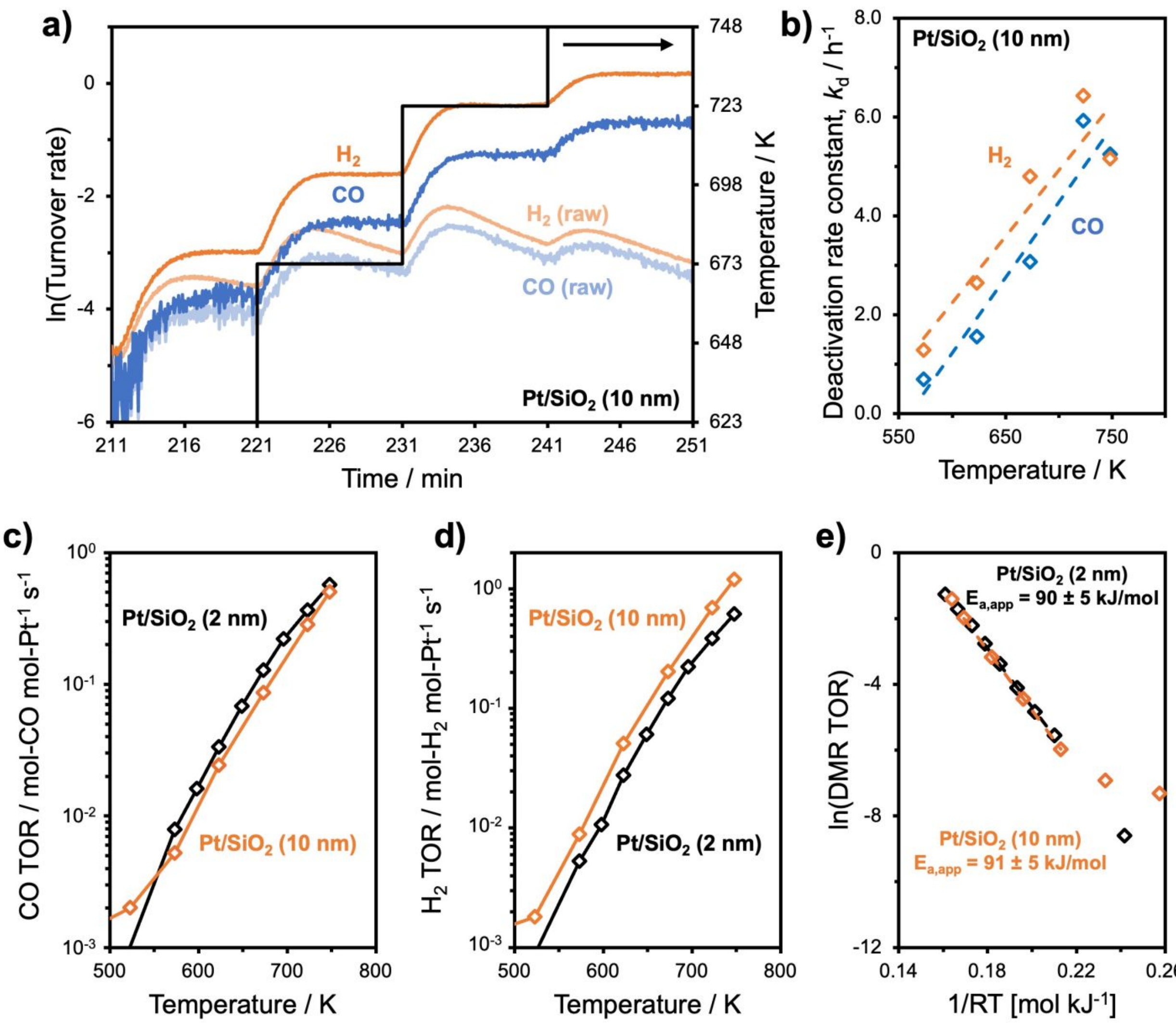

Figure S18: (a) As-measured turnover rates for CO (light blue) and H_2 (light orange) and deactivation-adjusted turnover rates for CO (blue) and H_2 (orange) as a function of time on stream while varying temperature (black, right axis) during thermal DMR on Pt/SiO_2 (10 nm) and (b) the corresponding extracted deactivation rate constants for CO and H_2 as a function of temperature. Steady-state thermal turnover rates for (c) CO and (d) H_2, and (e) Arrhenius plots for thermal DMR on Pt/SiO_2 (2 nm) (black) and Pt/SiO_2 (10 nm) (orange). Reaction conditions: $P_{CH4} = P_{CO2} = 6$ Torr, $P_{Ar} = 3$ Torr, and 100 sccm total flow.

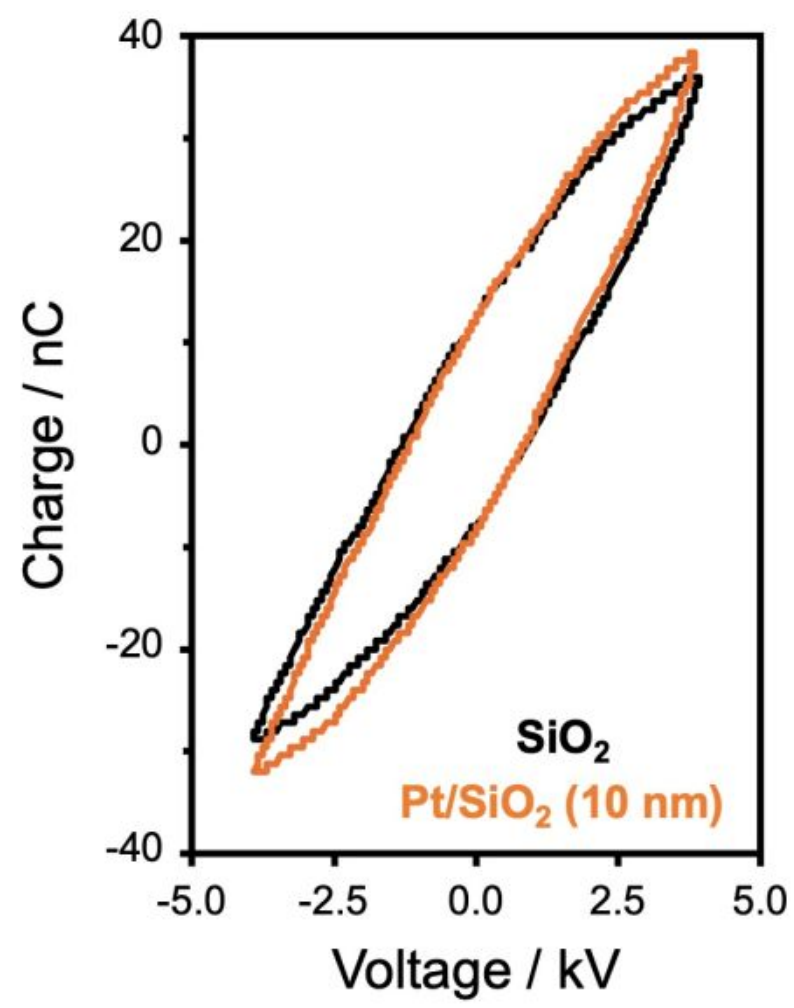

Figure S19: Lissajous (Q–V) curves for metal-free SiO_2 (black) and 10 nm Pt/SiO_2 (orange) during plasma-assisted DMR. Reaction conditions: T = 473 K, $P_{CH4} = P_{CO2}$ = 6 Torr, P_{Ar} = 3 Torr, and 100 sccm total flow at an applied voltage of 8 kV.

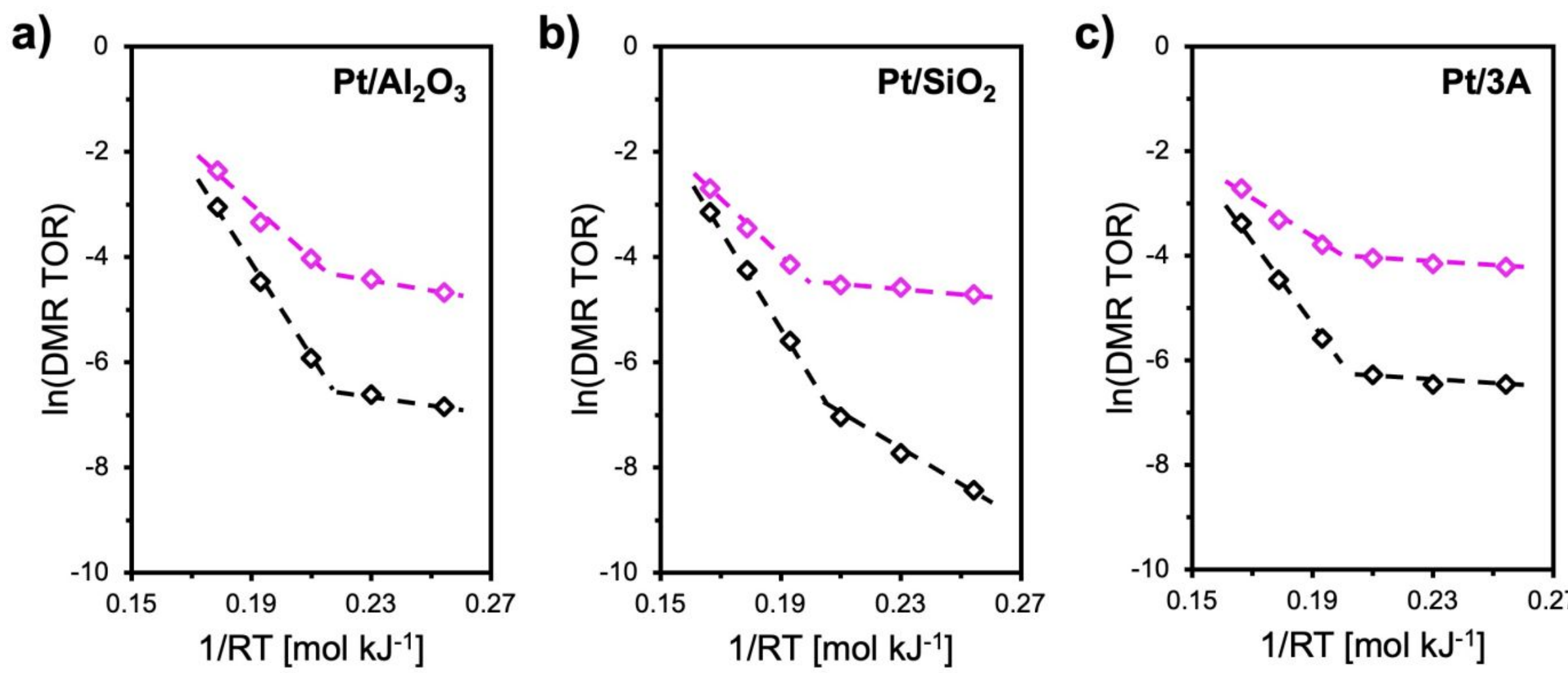

Figure S20: Arrhenius plots for DMR with (pink) and without (black) 8-kV plasma for (a) Pt/Al_2O_3, (b) Pt/SiO_2, and (c) Pt/3A. Reaction conditions: $P_{CH4} = P_{CO2}$ = 6 Torr, P_{Ar} = 3 Torr, and 100 sccm total flow.

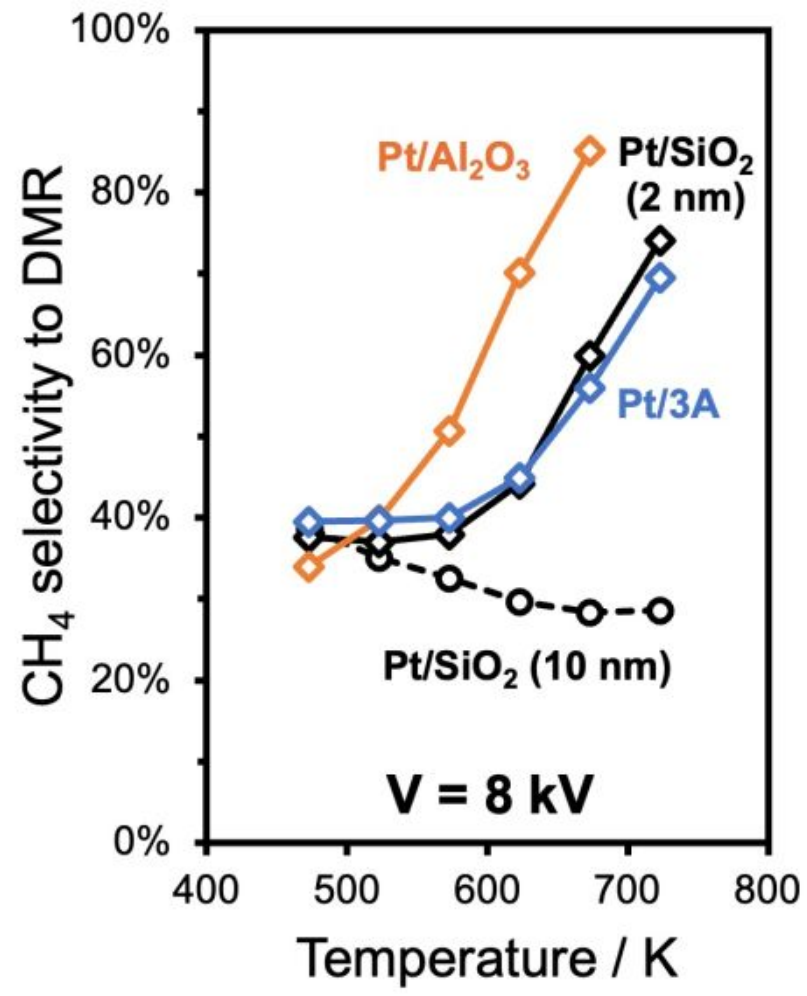

Figure S21: CH_4 selectivity to DMR during plasma-assisted DMR on Pt/Al_2O_3 (orange), Pt/SiO_2 (2nm, solid black), Pt/3A (blue), and Pt/SiO_2 (10 nm, dashed black) as a function of temperature. All measurements were recorded at 8 kV applied voltage. Reaction conditions: $P_{CH4} = P_{CO2} = 6$ Torr, $P_{Ar} = 3$ Torr, and 100 sccm total flow.

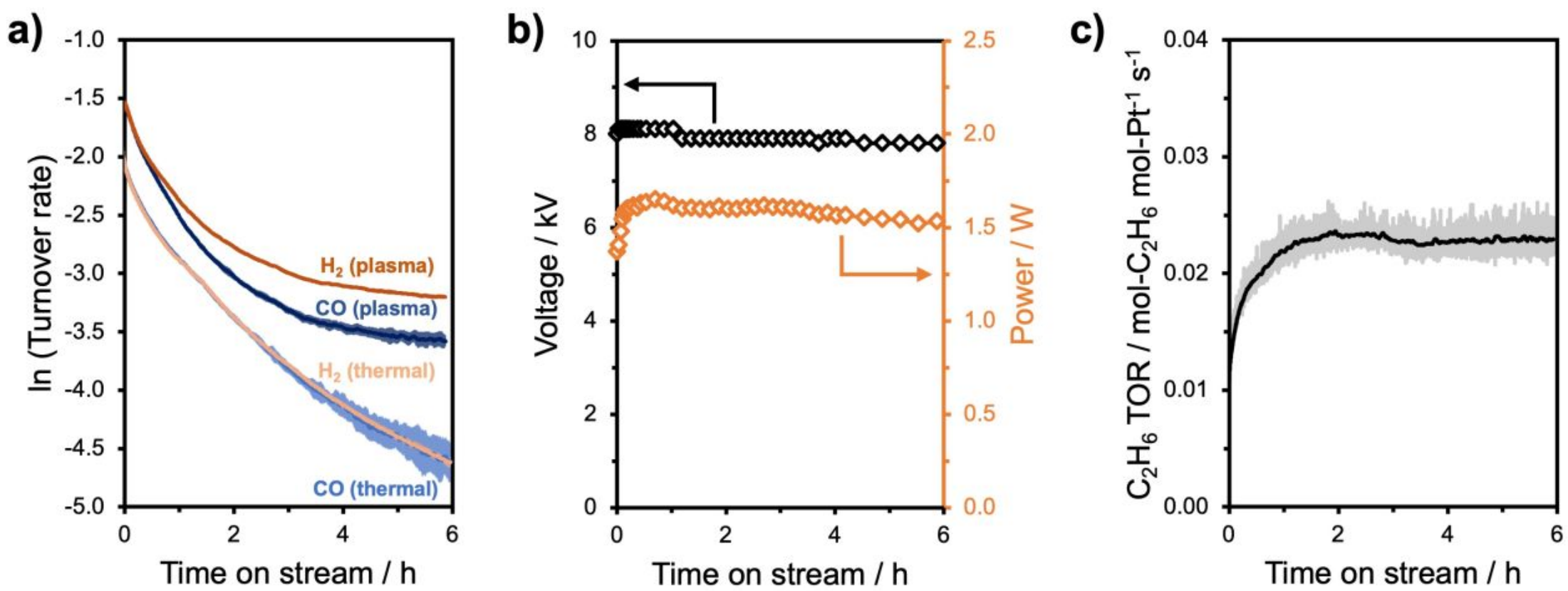

d)

Condition	$k'_{d,CO}$ / h^{-1}	$k'_{d,H2}$ / h^{-1}
Thermal (kinetic measurement)	0.82	0.88
Thermal (stability measurements)	0.86	0.87
Plasma (stability measurements)	0.75	0.50

Figure S22: Stability of Pt/SiO_2 during thermal and plasma-assisted DMR over 6 h time on stream at 723 K. (a) Natural logarithm of CO and H_2 turnover rates (TORs) during thermal (light blue and light orange, respectively) and plasma-assisted (dark blue and dark orange, respectively) DMR. Solid lines represent 5 min moving averages and shaded regions represent ±1 standard deviation within the moving window. (b) Applied voltage (black) and plasma power (orange) during plasma-assisted DMR. (c) C_2H_6 TOR during plasma-assisted DMR. The solid line represents a 5 min moving average and the shaded region represents ±1 standard deviation within the moving window. (d) Deactivation rate constants for CO and H_2 from thermal kinetic measurements and thermal and plasma-assisted stability measurements. For the stability measurements, deactivation rate constants were obtained over intervals corresponding to comparable extents of rate loss. Reaction conditions: 723 K, $P_{CH4} = P_{CO2} = 6$ Torr, $P_{Ar} = 3$ Torr, and 100 sccm total flow; an applied voltage of 8 kV was used during plasma-assisted DMR.

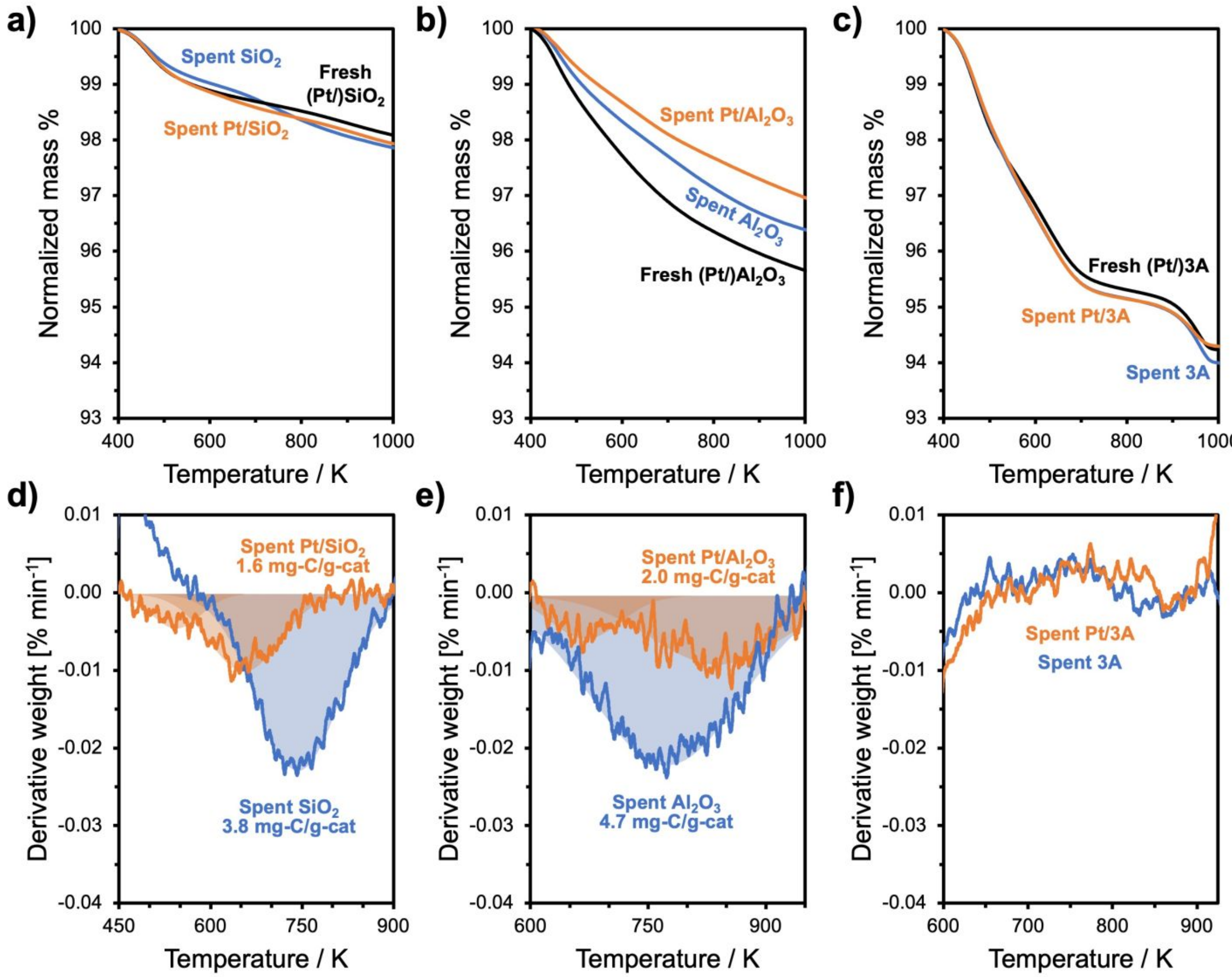

g)

Catalyst	Coke content [mg-C g-cat^{-1}]	SA-norm. coke content [µg-C m^{-2}]	Cumulative CH_4 converted [mmol]	Coke deposited/CH_4 converted [mol-C mol-$CH_4$$^{-1}$]
SiO_2	3.8	14	0.54	0.485
Pt/SiO_2	1.6	6	7.96	0.014
Al_2O_3	4.7	17	0.71	0.255
Pt/Al_2O_3	2.0	7	9.70	0.008
3A	n.q.	n.q.	0.46	n.q.
Pt/3A	n.q.	n.q.	3.69	n.q.

Figure S23: Normalized mass profiles during oxidative TGA of fresh and spent (a) (Pt/)SiO_2, (b) (Pt/)Al_2O_3, and (c) (Pt/)3A following plasma-assisted DMR. (d–f) Corresponding difference DTG profiles obtained by subtracting the fresh material from the respective spent material profiles. Shaded regions in (d–e) indicate mass loss features used to quantify deposited carbon. (g) Summary of coke content, BET surface area-normalized coke content, cumulative CH_4 converted, and the molar ratio of coke deposited to CH_4 converted for each spent material. *n.q.*, not quantified.

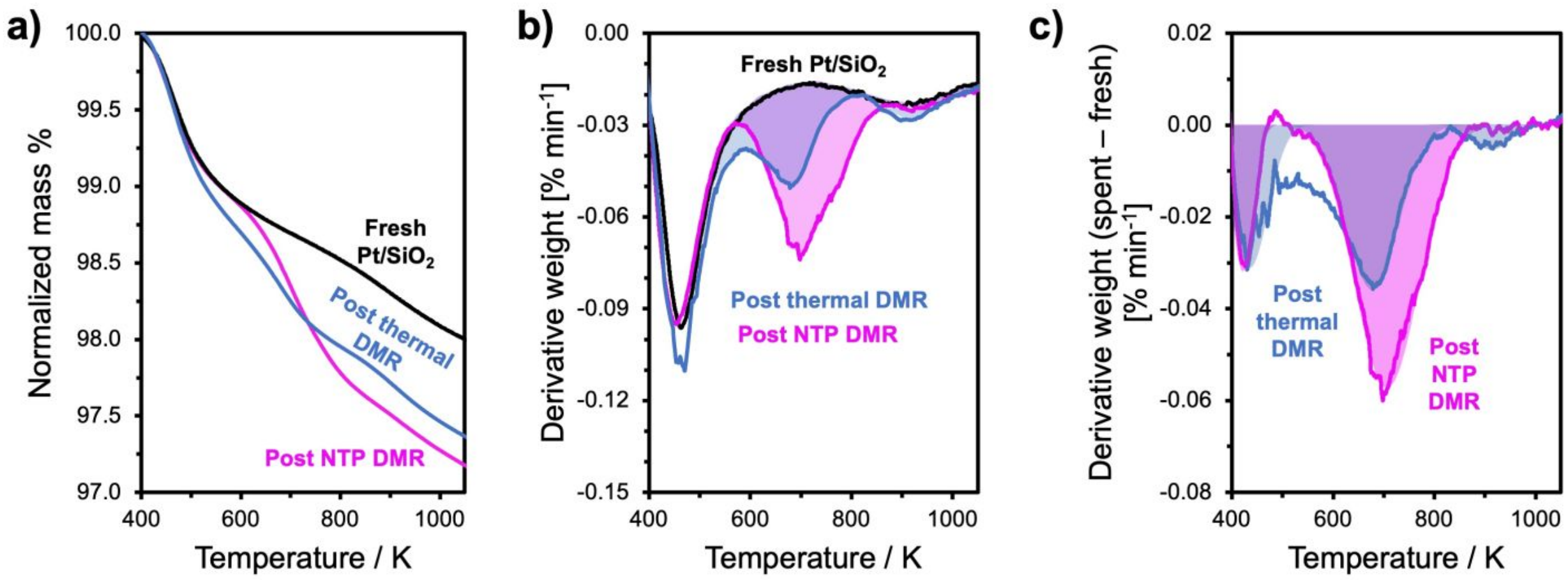

d)

Sample	T_{peak} [K]	Mass loss [wt.%]	Coke content [mg-C g-cat^{-1}]	Cumulative CH_4 converted [mmol]	Coke deposited/CH_4 converted [mol-C mol-$CH_4$$^{-1}$]
Thermal DMR	448	0.27	8.4 ± 0.6	8.1 ± 0.2	**0.072 ± 0.005**
	666	0.53			
	911	0.04			
NTP-assisted DMR	427	0.13	10.1 ± 0.4	29.1 ± 0.3	**0.024 ± 0.001**
	707	0.88			

Figure S24: Oxidative thermogravimetric analysis of Pt/SiO_2 before and after 6 h of thermal and NTP-assisted DMR at 723 K. (a) Normalized mass profiles and (b) corresponding derivative weight profiles of fresh Pt/SiO_2 (black) and Pt/SiO_2 following thermal (blue) and NTP-assisted (pink) DMR. The shaded regions highlight the main mass-loss features. (c) Difference DTG profiles obtained by subtracting the fresh Pt/SiO_2 profile from those of the spent catalysts, with shaded regions indicating the Gaussian-fitted mass-loss features used for coke quantification. (d) Peak temperatures and integrated mass losses, total coke contents, cumulative CH_4 converted during the 6 h experiments, and molar ratios of coke deposited to CH_4 converted. Uncertainties reflect TGA peak quantification, CH_4 conversion integration, and their propagation.

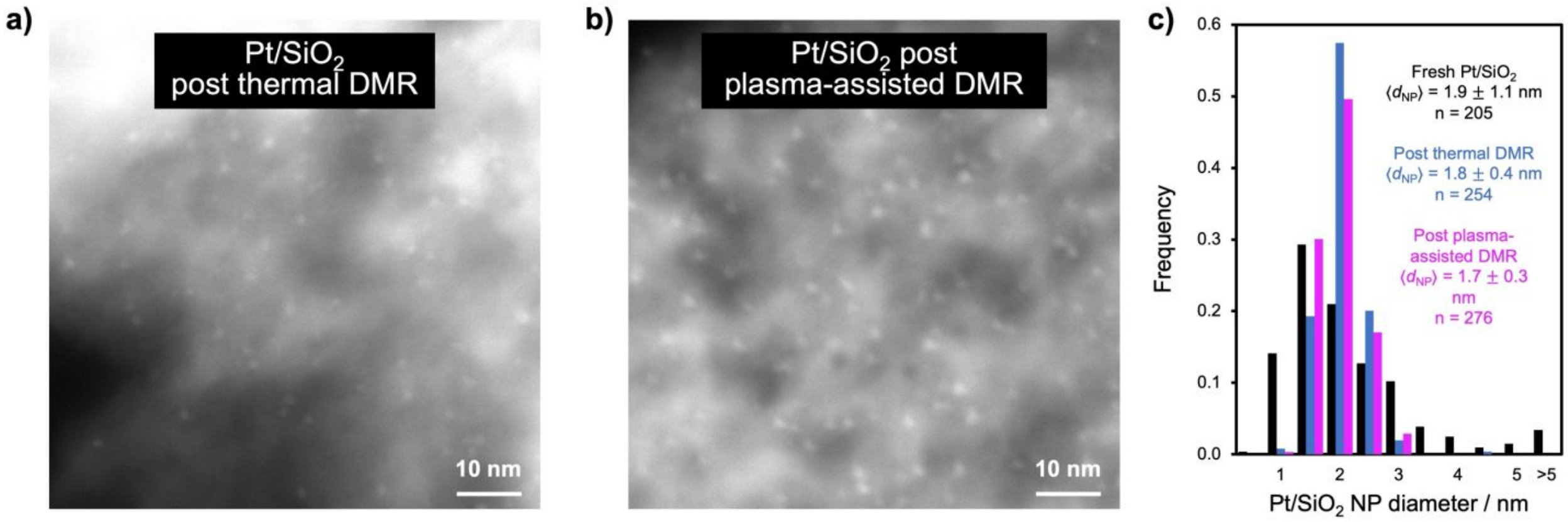

Figure S25: TEM micrographs of Pt/SiO_2 following 6 h of (a) thermal DMR and (b) plasma-assisted DMR, with (c) corresponding Pt nanoparticle size distributions, including fresh Pt/SiO_2.

Table S2: Pt nanoparticle sizes from TEM and Pt dispersions from CO-pulse chemisorption before and after plasma-assisted DMR on supported Pt catalysts.

Catalyst	Fresh NP size (nm, TEM)	Spent NP size (nm, TEM)	Fresh dispersion (CO-pulse)	Spent dispersion (CO-pulse)
1 wt.% Pt/Al_2O_3	1.8 ± 0.6	1.7 ± 0.7	0.59	0.61
1 wt.% Pt/SiO_2	1.9 ± 1.1	1.6 ± 0.6	0.40	0.34
1 wt.% Pt/3A	3.1 ± 4.5	3.1 ± 1.3	0.27	0.14
